# Students and brides: a qualitative analysis of the relationship between girls’ education and early marriage in Ethiopia and India

**DOI:** 10.1186/s12889-018-6340-6

**Published:** 2019-01-07

**Authors:** Anita Raj, Marissa Salazar, Emma C. Jackson, Natalie Wyss, Katherine A. McClendon, Aarushi Khanna, Yemeserach Belayneh, Lotus McDougal

**Affiliations:** 1 Department of Education Studies, Division of Social Sciences, University of California San Diego, 9500 Gilman Drive, La Jolla, CA 92093 USA; 2 Center on Gender Equity and Health, Department of Medicine, University of California San Diego, 9500 Gilman Drive, La Jolla, CA 92093 USA; 3 Center for Innovative Public Health Research, 555 N. El Camino Real, San Clemente, CA 92672 USA; 4 Hubert Department of Global Health, Rollins School of Public Health, Emory University, 1518 Clifton Road, Atlanta, GA 30322 USA; 5 School of Nursing, University of California, San Francisco, 2 Koret Way, San Francisco, CA 94143 USA; 6 Population and Reproductive Health Program, David and Lucile Packard Foundation, New Delhi, India; 7 Population and Reproductive Health Program, David and Lucile Packard Foundation, Addis Ababa, Ethiopia

**Keywords:** Early marriage, education, adolescent health, gender roles, Ethiopia, India

## Abstract

**Background:**

Early marriage (< 18 years) is associated with education cessation among girls. Little research has qualitatively assessed how girls build resiliency in affected contexts. This study examines these issues in Oromia, Ethiopia and Jharkhand, India among girls and their decision-makers exposed to early marriage prevention programs.

**Methods:**

Qualitative interviews were conducted with girls who received the intervention programs and subsequently either a) married prior to age 18 or b) cancelled/postponed their proposed early marriage. Girls also selected up to three marital decision-makers for inclusion in the study. Participants (*N* = 207) were asked about the value and enablers of, and barriers to, girls’ education and the interplay of these themes with marriage, as part of a larger in-depth interview on early marriage. Interviews were transcribed, coded, and analyzed using latent content analysis.

**Results:**

Participants recognized the benefits of girls’ education, including increased self-efficacy and life skills for girls and opportunity for economic development. A girl’s capacity and desire for education, as well as her self-efficacy to demand it, were key psychological assets supporting school retention. Social support from parents and teachers was also important, as was social support from in-laws and husbands to continue school subsequent to marriage. Post-marriage education was nonetheless viewed as difficult, particularly subsequent to childbirth. Other noted barriers to girls’ education included social norms against girls’ education and for early marriage, financial barriers, and poor value of education.

**Conclusion:**

Social norms of early marriage, financial burden of school fees, and minimal opportunity for girls beyond marriage affect girls’ education. Nonetheless, some girls manifest psychological resiliency in these settings and, with support from parents and teachers, are able to stay in school and delay marriage. Unfortunately, girls less academically inclined, and those who do marry early, are less supported by family and existing programs to remain in school; programmatic efforts should be expanded to include educational support for married and childbearing girls as well as options for women and girls beyond marriage.

**Electronic supplementary material:**

The online version of this article (10.1186/s12889-018-6340-6) contains supplementary material, which is available to authorized users.

## Background

Early marriage, defined as marriage before the age of 18, is a pervasive health and human rights issue that disproportionately affects women and girls [1]. Globally, 15 million girls are married by age 18 each year; by the year 2030, 950 million girls will have been married as children [1]. Early marriage is associated with serious adverse health and social outcomes, including compromised sexual, reproductive, and maternal health, increased risk of depression and suicidality [2–9], greater risk of intimate partner violence [4–8], decreased social and physical mobility, and decreased autonomy in decision-making within and outside of the household [10, 11]. Early marriage also compromises girls’ ability to attend school post-marriage, exposing them to an array of adverse social and health outcomes associated with education cessation [10–17].

Though early marriage is declining globally, prevalence remains highest in Sub-Saharan Africa and South Asia [1]. In India, 27% of 20–24 year old females are married by age 18, and 7% are married by age 15 [18]. In Ethiopia, 40% of 20–24 year old females are married by age 18, and 14% are married by age 15 [19]. Over the last ten years, early marriage has declined by 19% in India and by 9% in Ethiopia [18, 19]. While this progress is commendable, the rate of progress is inadequate when more than one in four girls in India and two in five girls in Ethiopia may be subject to this harmful practice and its deleterious social and health effects.

Research has identified that promotion of girls’ education is a key means of reducing early marriage in many contexts [20–22]. While girls not in school may be more vulnerable to early marriage, early marriage itself may serve as a barrier to post-marriage school attendance due to early childbirth and related child-care responsibilities [12], and/or restriction of school attendance by girls’ husband or in-laws [17, 23]. Hence, the association between education and early marriage is likely bidirectional [10, 16, 22, 24–27]. Reduced economic self-sufficiency as a result of education cessation and subsequent early marriage is also of particular concern in contexts where the burden of early marriage is highest [1, 10, 11, 13, 14, 16, 22, 28–34]. School cessation limits economic empowerment, specifically in the ability to earn an independent income, which is often further constrained within the context of early marriage [10, 11, 14]. On the other hand, educational attainment increases economic self-sufficiency among women and girls, and is associated with less reliance on male partners, as well as increased self-efficacy [20, 32, 35, 36]. The consequences of education cessation are similar to that of early marriage and include reduced access to sexual and reproductive health education and services, social isolation from peers and mentors, and decreased social mobility underscored by economic vulnerability [11, 22, 34, 37, 38].

Given the well-documented association between education cessation and early marriage, many programmatic efforts now focus on keeping girls in school in an effort to reduce early marriage [20, 22, 39]. Several programs aimed to increase girls’ school attendance have also shown decreases in early marriage [40–43]. However, investigation of this relationship has been primarily quantitative in nature; little work has examined how girls and communities perceive the costs and benefits of girls’ education relative to early marriage and what norms reinforce these perceptions [10, 22, 44]. The pathways connecting girls’ education and delayed marriage are rooted in social norms and values related to girls and gender equality, therefore a deeper understanding of this interplay is needed to comprehensively address these issues [8].

One lens through which we can understand how adolescents can challenge these norms is that of Resiliency Theory, which takes a strength-based approach to posit that youth facing serious stressors and barriers are able to overcome obstacles and succeed with assets or resources available at the contextual, social or individual level [45]. Such assets and resources can include community programs and family support for education, as well as self-efficacy and aspiration at the individual level of the girl. Through this study, we sought to gain insight into obstacles and resilience related to the relationship between girls’ education and early marriage in Oromia, Ethiopia and Jharkhand, India, where the practice of early marriage remains common [18, 19]. This study includes data from both girls and their key marital decision-makers, as girls in these contexts are often unable to decide unilaterally when and whom to marry.

## Methods

### Study setting

This study uses data collected in Oromia, Ethiopia and Jharkhand, India. Oromia has a population of 27 million people, 88% of whom live in rural areas [46]. Educational attainment is low; more than half of women (51%) have had no formal education, and only 12% have secondary or higher education [19]. Amongst women aged 15–49 years old, 33% were employed in the past 12 months, compared to 92% of male counterparts [19]. The median age of marriage among 20–24 year olds was 18.2 [19, 47]. Similarly, the Indian state of Jharkhand has a population just under 33 million, with 76% residing in rural areas [48]. Educational attainment in Jharkhand is also low; 37% of women have no formal education, though more than half (52%) have secondary or higher education [18, 47]. Thirty-two percent of women aged 15–49 years old were employed in the last 12 months, compared to 97% of male counterparts, and the median age at first marriage among 20–24 year old females was 19.0 [18].

### Study design and sample

Data from the current study were collected as part of a larger program aimed at reducing early marriage and improving reproductive and sexual health among adolescent girls. While implementation of the program differed slightly, program aims were consistent. The Oromia Development Association Comprehensive Adolescent/Youth Sexual and Reproductive Health Project (“ODA”) is a school-based program which began in 1993 in Oromia, Ethiopia. ODA is implemented by teachers and operates both within schools and through community outreach. Project RISHTA, first implemented in 2001 in Jharkhand, India, is a community-based program delivered by trained youth leaders. Both programs incorporate instruction on adolescent marriage, the health effects of early pregnancy and childbirth, family planning and contraception, and vocational training.

In 2014, research staff conducted qualitative interviews with program participants aged 13–24 years and up to three of their marital decision makers to investigate the underlying structures that lead to delayed early marriage, specifically the complexities of marital decision-making. To be eligible for an interview, participants had to be female ODA/Project RISHTA participants who either: a) married prior to the age of 18 (“girl married <18”), or b) were able to cancel/postpone marriage until age 18 or later (“girl able to cancel/postpone marriage”). Interviewed girls were also asked to identify up to three marital decision makers to participate in the study. Participants in Ethiopia were recruited in two of the eight “woredas” (districts) that originally implemented the ODA project, resulting in a sampling frame of 124 primary schools in Oromia. Teachers, community health workers, and program staff identified potential participants, and interviewers subsequently recruited participants directly from their homes. Similar recruitment methods were used in India; a sampling frame of 32 villages within the Saraikela Kharsawan district of Jharkhand were identified, a list of all eligible participants was created, and interviewers went to women and girls’ homes to invite them to participate. In the Indian sample, preference was given to women and girls whose marriages or proposals were most recent.

### Procedure

Trained research staff conducted semi-structured, audio-recorded interviews lasting approximately 45 minutes with ODA/Project RISHTA participants (*N* = 104) and up to three of their marital decision makers (*N* = 161). In Ethiopia, 23 women and girls married < 18 and 42 of their marital decision makers were interviewed, as well as 27 women and girls able to cancel/postpone marriage < 18 and 59 of their marital decision makers. In India, 25 women and girls married < 18 and 29 of their marital decision makers were interviewed. Additionally, 29 women and girls able to cancel/postpone marriage < 18 and 31 of their marital decision makers were interviewed. Further details on study procedures are discussed elsewhere [49–51].

Interviews were de-identified, transcribed and translated into English. Following translation, we excluded 58 interviews from analyses due to poor interview quality or failure to meet eligibility requirements (e.g., duplicate interviews, and unmatched decision-maker participants). Excluded interviews were primarily (78%) from Ethiopia, though final samples were comparable across groups. Our final analytic sample of *n* = 207 included:

- 21 girls with canceled/delayed early marriages in Ethiopia and 41 of their marital decision-makers
- 23 girls married as minors in Ethiopia and 21 of their marital decision-makers
- 24 girls with canceled/delayed early marriages in India and 27 of their marital decision-makers
- 25 girls married as minors in India along with 25 of their marital decision-makers.

### Interview Questions

The in-depth interviews aimed to understand participants’ perceptions of ODA and Project RISHTA, and their views on early marriage and education (see Additional file 1). Questions related to education assessed participants’ attitudes towards education, their perceptions of the benefits and disadvantages of education, gender norms related to girls’ education, and educational attainment post marriage and post childbirth. Sample questions included: “*Up to what level should a girl continue her education before getting married?*” *“What do you think about girls’ education?”* and “*Do you think girls should go to school like boys do?*”

### Data Analysis

Two PhD level social science and public health researchers oversaw all data analysis for this study, and, in partnership with three trained research assistants, reviewed 20% of all interviews by type of interviewee. A latent content analysis was used to develop a coding structure with themes and sub-themes under a series of domains corresponding with content foci of interview questions (e.g., early marriage, marital choice and decision-making, education) [52, 53]. A final coding scheme was created based on emergent theme identification and coder agreement. Seven trained research assistants coded and analyzed all data using Atlas.ti. Inter-coder reliability was assessed using Cohen’s kappa> 0.9.

Current analyses focus on findings related to education, which generated the following themes: perceived benefits and disadvantages of girls’ education; enablers and barriers to girls’ continuation of secondary education; enablers and barriers to girls’ continuation of secondary education subsequent to marriage. Subthemes and sub-subthemes were developed from these themes, providing a hierarchical analysis of themes constructed into dendritic trees. Further detail on our coding process can be found in our previous publications [49–51]. Using resiliency theory as our framework, we again reviewed themes and subthemes to reflect on enablers supporting girls’ education, and to profile social and cognitive assets and resources more directly [45].

## Results

### Participant Characteristics

Participants (*N* = 207) from the current study ranged in median age from 14 to 19 years among girls, and 26–39 years among decision-makers (Table 1). The relationship between decision-makers and girls varied by country and marital status. Among girls in Ethiopia whose marriages were postponed/cancelled, the majority of decision-makers were participants’ teachers, followed by father, mother, and other relatives (e.g., siblings, aunts/uncles, and grandparents), whereas decision makers of married participants were generally their parents. In India, decision makers of girls whose marriages were postponed/cancelled were primarily their parents, RISTHA staff, and other relatives, whereas decision makers of married participants were mostly their husbands, mothers, fathers and other relatives.

**Table 1:** Demographic summary of participants (*n* = 207)

	Ethiopia				India			
	Married < 18		Cancelled/postponed marriage		Married < 18		Cancelled/postponed marriage	
	Girl	Decision-maker	Girl	Decision-maker	Girl	Decision-maker	Girl	Decision-maker
Total n(%)	23 (21.7%)	21 (19.8%)	21 (19.8%)	41 (38.7%)	25 (24.8%)	25 (24.8%)	24 (23.8%)	27 (26.7%)
Median age (IQR)	18 (16–18)	38 (30–45)	14 (14–15.5)	26 (24–30)	18 (18–20)	27 (23–41)	19 (18–20.25)	39 (32.5–45)

#### THEME 1. Benefits and Disadvantages of Girls’ Education

##### Benefits of Girls’ Education

**Fig. 1:**
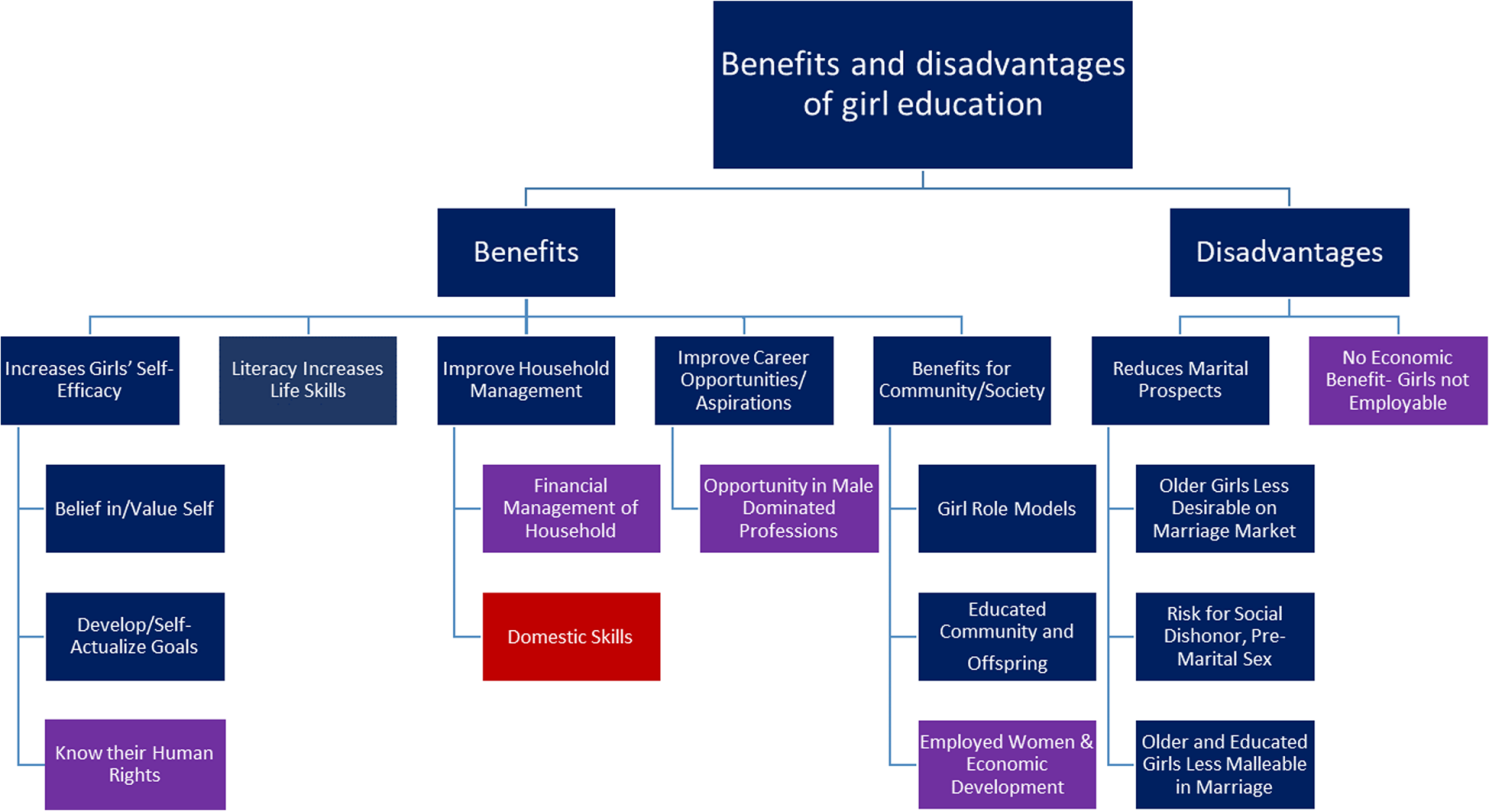
Benefits and Disadvantages of Girls Education: *Note: Blue indicates findings reflect Ethiopia and India. Purple indicates findings only reflect Ethiopia. Red indicates findings only reflect India*

In both Ethiopia and India, participants had primarily positive views regarding girls’ educational attainment, and participants reported benefits directly for girls and the families and children they may have, as well as benefits for the larger community and society (Fig. 1). Benefits directly related to girls included improved life skills, domestic capacities related to both household management and childrearing, and economic capacities including financial management of households and potential career development. These benefits were recognized as bringing value to the family and the community as a whole.

*Education Increases Girls’ Self-Efficacy*. Participants in both India and Ethiopia noted the value of education in terms of supporting girls to have increased perceived and actual self-efficacy to be able to value themselves, develop and self-actualize their own goals, and manage their lives.

*“If [a girl is] not educated, she may not be able to identify what will help or harm her. She has no awareness about the socioeconomic benefits [of education]. [If a girl is not educated,] it makes her think that she is less than boys.”* **[Girl able to cancel/postpone marriage, Student, age 13–17, Ethiopia]**

*“The future for girls who are educated is brighter because they have more knowledge. The girls who are not educated only know what they see on television or what others tell them.”* **[Female decision-maker for girl able to cancel/postpone marriage, Tata Steel Family Initiative Foundation, Sarna, age 35–49, India]**

*“The educated girls make their own future. The uneducated are like “bandhi hui gai” (like a cow tied to a pole) who is dependent on in-laws and her husband.”* **[Girl married <18, Student, Sarna, age 18–24, India]**

Additionally, participants in Ethiopia emphasized the value of education and safety to pursue education as human rights, and the role of education in helping girls recognize their human rights.

*“Students know the basic human and democratic rights... In previous regimes, girls were not deciding their rights but now they have right to decide. Now, she has learned everything about the constitution and rights... With coordination of school administration and the community, efforts are underway to respect her rights.”* **[Male decision maker for girl able to cancel/postpone marriage, Teacher, Protestant, age 18–24, Ethiopia]**

*“They (girls) have equal rights with boys to continue their education. The problem is with the community. There are only 44 students enrolled in our school this year while there are a lot of girls in the community. The society doesn’t want to send their girls to school.”* **[Male, decision maker for girl able to cancel/postpone marriage, Teacher, Orthodox, age 25–34, Ethiopia]**

*“When I was attending the lower class, meaning from grade 1-3, everybody was abusing us on our way to and from school. But recently, after gender equality has been ensured and women’s right has legally been protected, I recognized that we have equal rights. So, no one can abuse me like that. There is also a time when we legally defended ourselves from those abusive men. There is also a time I told my parent and defended myself.”* **[Girl able to cancel/postpone marriage, Student, Muslim, age 13–17, Ethiopia]**

*Education Increases Literacy, Supporting Girls’ Life Skills*. Decision-makers also noted the importance of literacy gained in schools in allowing girls to participate in and navigate life more effectively and less vulnerably.

*If [girls] don’t learn, they are going to be illiterate like we are.* [**Female decision maker for girl married <18, Farmer, Muslim, age 35-49, Ethiopia]**

*“…Even for travelling, [an education is needed to do things like] reading the road signs and the bus numbers* ***.”*** **[Male decision maker for girl able to cancel/postpone marriage, Health Worker, Hindu, age 25–34, India]**

The value of literacy was also tied to marital prospects.

*“The literate girls are more appreciated by people and they get good marriage proposals. They are more intelligent and street-smart. The illiterate girls are very gullible.”* **[Female decision maker for girl married <18, Housewife, Sarna, age 25–34, India]**

*Education Increases Girls’ Domestic Capacities – Household Management and Childrearing*. Related to life skills, but more gendered in nature, participants described education as increasing girls’ domestic capacities for family life, both in terms of managing a household and being a good mother. These concepts were noted in both countries, but more often in India.

*“[An educated girl] can give proper upbringing and education to her child since males don’t stay at home and educate the children, who are the future of the family, community & village. [Educating the children] is the sole responsibility of the female.”* **[Girl married <18, Student, Hindu, age 18–24, India]**

*“Yes, it is important to educate girls the way boys are educated, as it makes the brain sharper and can be helpful in so many ways, including managing a family, educating one’s own children, managing household affairs, and even earning [money]”* **[Girl married <18, Housewife, Sarna, age 18–24, India]**

*“In education, one who cannot learn cannot change oneself, can keep her house clean, can agree with their partner* ***.*** *So, if marriage delays, the girls can get a chance of completing their education and can get those advantages.”* **[Male decision maker for girl able to cancel/postpone marriage, Civil Servant, Muslim, age 25–34, Ethiopia]**

Participants in India further noted that this increased domestic capacity could reduce risk of conflict with husbands and in-laws.

*“It is good to educate girls like the boys [are educated]. If a girl is educated, she will be able to manage her house and family well and chances of conflicts with husband and in-laws will reduce substantially. [An educated girl] understands issues like the importance of a small family with one or two children, she is in a better position to educate her child, and can even earn a good amount [of money] by working.”* **[Female decision maker for girl married <18, SHG/Peer Educator with Project RISHTA, Sarna, age 25–49, India]**

Conversely, girls denied education due to early marriage recognized the loss, and worried about how it compromised their ability to support their children’s educational development.

*“Yes, it is important for the girls to go to school like boys do. I am not educated and I feel restricted in many things. Like I cannot read and write, and now that my son is growing, I won’t be able to teach him either. I will be completely dependent on the school [to teach him].”* **[Girl married <18, Housewife, Sarna, age 18–24, India]**

*Education Increases Girls’ Economic Capacities – Household Financial Management and Career Aspirations and Opportunities*. In Ethiopia, though not India, participants described how girls’ education could increase financial management skills in ways that could be applied to the household and could help ensure husbands did not misuse household funds.

*“Upon getting [an] education, girls learn how to keep their child, themselves, and their homes clean. Yes, girls must learn, since it helps them with home management. We can get knowledge on how to save money and wash home tools...”* **[Girl married <18, Housewife, Muslim, age 18–24, Ethiopia]**

*“In terms of economy, [an educated woman] will work with her husband because she has a good experience. If he tries to spend their wealth, she will force him to save. Since she has experiences [with education] she will advise him on how to raise their children, how to make a living, and how to save money.”* **[Girl able to cancel/postpone marriage, Student, Muslim, age 13–17, Ethiopia]**

Discussion in both India and Ethiopia touched on girls’ education as a driver of girls’ career aspirations and opportunities. However, in India girls discussed careers oriented in traditionally female roles (e.g., teacher, cook, embroidery work), recognizing that some careers require more education than others.

*“I would like to study until class 8 or class 10. [When I am done with school,] I would like to do embroidery work and stitching etc.”* **[Girl able to cancel/postpone marriage, Muslim, age 18–24, India]**

*“I want to do BA, MA and then become a teacher. I love studying and teaching.”* **[Girl able to cancel/postpone marriage, Student, Hindu, age 13–17, India]**

Participants from India described female employment as benefitting the family as a whole, and as protection for girls who do not marry.

*“If a girl is educated, the entire family is educated. She can go for a well-paid job and is in a better condition to bear the expenditures of a family. The family [of an educated girl] is more mature and financially better off.”* **[Girl able to cancel/postpone marriage, Student, Hindu, age 18–24, India]**

At the same time, some recognized that they may have to contend with family or in-laws’ attempts to impede their career aspirations, despite these recognized benefits. However, they felt able to achieve their goals despite any resistance from family.

*“I would like to complete my education, do B. A. and then I want to become a teacher. There may be some problems and the in- laws may not allow [me to do this], but if one wants [to do something] nobody can stop [them].”* **[Girl able to cancel/postpone marriage, Student and Housewife, Hindu, age 18–24, India]**

In contrast, participants in Ethiopia discussed more diverse careers for girls that were not limited to predominantly female occupations, and even described career opportunity as a means for girls’ independence and control over decisions regarding their life course. Employment was tied to increased agency and freedom, as opposed to viewing employment solely as a means of economically contributing to the family or to being a more appealing partner.

*“But as my own plan I need to continue my education so that I can be employed at a government institution. My parents are telling me to marry after grade 10. So, I am planning to push forward, to continue until university. I need to be a doctor.”* **[Girl able to cancel/postpone marriage, Student, Muslim, age 13–17, Ethiopia]**

*A girl should not marry until she complete her education and get her own job. Because she has to get job and be independent.* **[Girl able to cancel/postpone marriage, Student, Muslim, age 13-17, Ethiopia]**

*“Economically speaking, [an educated girl] can manage her life very well; the family will not be disturbed; at this stage this student or girl can be independent... Socially, she can live without support [due to her own employment].”* **[Male decision maker for girl able to cancel/postpone marriage, Teacher, Orthodox, age 25–34, Ethiopia]**

*“My idea is to reach a high position and create my own job. If I complete the university level, then I will get a better job. Then, since I will be making my own income, I can also choose my partner. Or if I don’t get married, I can live on my own income.*” **[Female decision maker for girl able to cancel/postpone marriage, Student, Muslim, age 13-17, Ethiopia]**

*Educating Girls Yields Benefits for the Community and Society*. Participants in both countries described how girls’ education positively shapes community and society in a number of ways. First, educated girls who delay marriage become community role models to support these changes for other girls and families in the community.

*“So, when I say that education is necessary for girls, [I mean that educated] girls become models for the community in eliminating early marriage. Therefore, education plays a significant role in transforming the lives of girls and the community in general. Girls must continue with their education to the higher level and be a role model for the community. That is why we are encouraging girl students here and mention cases of academically successful girls”* **[Male decision maker for girl able to cancel/postpone marriage, Teacher, Protestant, age 18–24, Ethiopia]**

Second, as educated girls are better able to support stronger families and more educated children, they build a better educated society.

*“If a girl is educated, it is the entire society that bears fruit- if the girl is educated she will ensure that her children are educated, then the entire family is educated and finally that the whole society that is educated.”* [**Girl able to cancel/postpone marriage, Student Hindu, age 13–17, India]**

*“Educating a girl will bring a direct change in the society; teaching a single girl is said to be teaching the whole family. This doesn’t mean, however, the whole family learns directly. But, when a girl learns, she knows how to manage the house… and she knows how to wisely spend her money.”* **[Female decision maker for girl able to cancel/postpone marriage, Teacher, Orthodox, age 18–24, Ethiopia]**

Third, as girls become community and society leaders and contribute to the formal labor market, they can strengthen the community and economic development. This was particularly noted in Ethiopia.

*“Women are the main actors in the community. They move the economy. So, if they get an education, it enable[s] them to further strengthen their role in the community.”* **[Male decision maker for girl able to cancel/postpone marriage, Merchant, Muslim, age 18–24, Ethiopia]**

*“If girls are educated, they can lead the country and teaching them will play a great role in our country development...”* **[Female decision maker for girl able to cancel/postpone marriage, Teacher, Orthodox, age 25–34, Ethiopia]**

*“Education is more important for girls than boys because education empowers girls. It supports them to withstand the bad cultural practices against them. We know that girls were regarded as incapable and incompetent. But it is because of education that they have started to enjoy equal treatment with boys. So, if girls have access to education, they further get power and confidence.”* **[Male decision maker for girl able to cancel/postpone marriage, Farmer, Muslim, age ≥50, Ethiopia]**

#### Disadvantages of Girls’ Education

Few participants discussed disadvantages of education, but those who did described education as negatively affecting girls’ marital prospects, either because older and educated girls are less desirable on the marriage market or because of fear of dating relationships and pre-marital sex affecting her chances of marriage and family honor as a whole (Fig. 1).

*Girls should not learn like boys do, because if girls complete their education, they become older and they might not get a husband since their age of marriage will pass. It is difficult to learn after marriage because the society harasses the married girls,* **[Girl married <18, Student, Muslim, age 13-17, Ethiopia**

*“Usually boys leave their studies and start working in the fields (agriculture) or go out and start working in nearby mines. Now that boys are not highly educated, they do not want a girl more educated than them. These mindsets will take time to change.”* **[Male decision maker for girl married <18, Agricultural Worker, Sarna, age 25–49, India]**

*“The society thinks that, if a marriage is delayed until 18 years old, the girls would not get married since males do not want to marry those who are 18 years old. The society also thinks that when girls go to school, they can have romantic relations which are much forbidden.”* **[Male decision maker for girl able to cancel/postpone marriage, Civil Servant, Muslim, age 25–34, Ethiopia]**

*“There are social pressures and we as parents of a girl are afraid that she might not take a wrong step and tarnish the image of the whole family. But, I think if Rishta people were not there, I would have not thought of canceling/ postponing my daughter’s marriage.”* **[Male decision-maker for girl able to cancel/postpone marriage, Masonry, Sarna, age 35–49, India**

In Ethiopia, there was also discussion regarding the economic disadvantage of girls’ education, as post-education employment options for women and girls were too limited to justify the financial burden of schooling.

*“Girls think marriage is a very big deal and haven’t seen anyone who succeeded in education so they thought that this is her only choice and a good life is waiting for her in marriage.* ***”*** **[Male decision maker for girl able to cancel/postpone marriage, Teacher, Orthodox, age 25–34, Ethiopia]**

*“My father decided my marriage, he believed that education takes [you] nowhere. He said if educated, students come back and put pressure upon family. They go nowhere, therefore she must marry. My mother also said the same thing. I was not happy with the decision.”* **[Girl married <18, Housewife, Muslim, age 13–17, Ethiopia]**

*“They (my family) even discouraged me (from school), saying that education is not worth the cost of pens and exercise books. Educated individuals themselves are not getting jobs.”* **[Girl married < 18, Housewife, Muslim, age 18–24, Ethiopia]**.

#### THEME 2. Enablers and Barriers to Continuation/Completion of Secondary Education

**Fig. 2:**
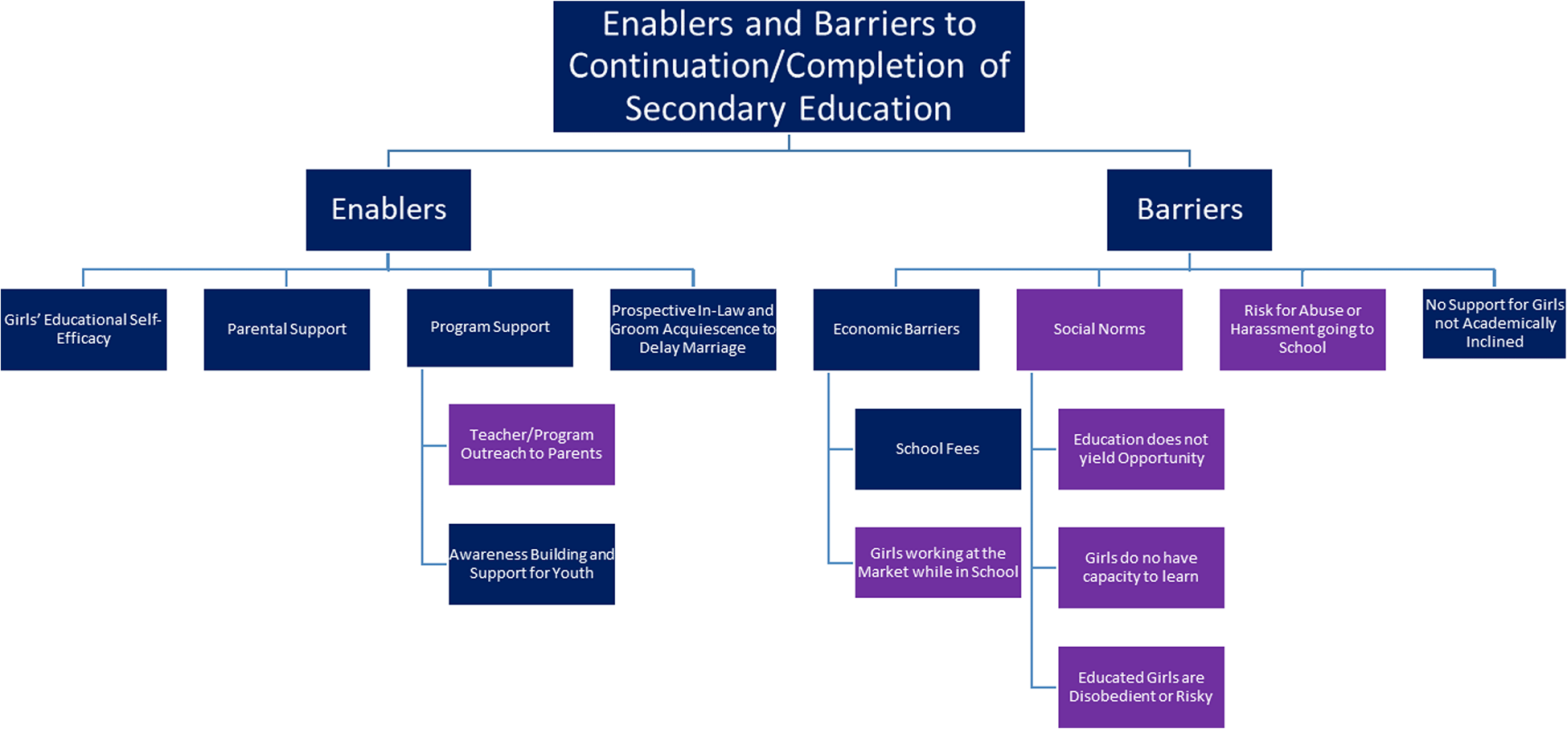
Enablers and Barriers to Continuation/Completion of Girls’ Secondary Education: *Note: Blue indicates findings reflect Ethiopia and India. Purple indicates findings only reflect Ethiopia*

In addition to perceptions on the benefits and disadvantages of education in general, participants also discussed specific factors that acted as enablers or barriers to completing secondary education (Fig. 2).

##### Enablers of Continuation/Completion of Girls’ Secondary Education

Enablers of continued education came from girls’ self-efficacy to voice desire for education despite social pressures to marry and withdraw from school, as well as parent and program support for girls’ continued education. In such contexts, prospective in-laws and grooms generally respected family decisions to keep girls in school and delay marriage.

*Girls’ Educational Self-Efficacy*. The most commonly noted enabler for girls’ continued education was the girl herself; if she was motivated to continue her education, this was commonly the justification to delay marriage.

*“It was she herself who first talked about the possibility of cancelling or postponing the marriage. She said she wants to continue her education. Then we all agreed.”* **[Male decision maker for girl able to cancel/postpone marriage, Farmer, Muslim, age 35–49, Ethiopia]**

*“A guy who needs to marry me told to his friend and his friend informed me. Then, I told him as I am not ready to get married before finish my university education. Then, he sends elders to my family and my family asked for my interest if I am willing or not. I answered as I am not ready for that and if they want they can marry him instead of me. For time is gold, I need to utilize for improving my life through education. I convinced my family refused to receive his proposal and my marriage has postponed.”* **[Girl able to cancel/postpone marriage, Student, Muslim, age 13–17, Ethiopia]**

*“I was the one who refused to get married at that age. I spoke to my mother about it and told her that I don’t want to marry and want to continue my education. She then informed my nani (Granny) and mausi (aunt).*” **[Girl able to cancel/postpone marriage, Muslim, age 18–24, India]**

Sometimes girls who were either forced into marriage or wanted to leave a marriage, used their self-efficacy to physically escape or seek legal aid.

*“Although I didn’t want too, the guy did some witch thing to me, which made me agree and marry. I didn’t want to quit learning so. I told my teachers that I will manage to learn in spite of getting married. After I stayed two weeks, I escaped and went back to home. My father talked to the teachers and I got back to school. And here I am now.”* **[Girl married < 18, Student, Muslim, age 18–24, Ethiopia]**

*“When I was grade six my family told me that they are going to give me (in marriage)… I told to my family that I have a wish for education. I told the police officer that my parents are not educated; they wanted to get me engaged. They arrested my parents, and I quit the school for that year. After that, I came here and started grade six.*” **[Girl able to cancel/postpone marriage, Student, Muslim, age 13-17, Ethiopia]**

*Parental Support*. Parents also reported a desire for girls to continue their education as a reason for preventing or postponing an early marriage, and showed support through several means including direct assistance with schoolwork.

*“My father canceled the marriage. I don’t know much, but I know that my father is very wise man. He wanted me to study and focus on my future. I believe that is why he has always encouraged me to study and as yet has not accepted any marriage proposal.*” **[Girl able to cancel/postpone marriage, Student, Sarna, age 18–24, India]**

*“I support her to focus on her education. I have no right to force her into marriage. The fact that she is doing* very *well in her education is what encouraged me on this decision. She respects me and I respect her decision.”* **[Female decision maker for girl able to cancel/postpone marriage, Farmer, Muslim, age 35–49, Ethiopia]**

*“Why did she decide to cancel or postpone? First, she is underage and second we thought her education should be a priority. She can get married anytime but she can’t catch the education.”* [Female decision maker for girl able to cancel/postpone marriage, teacher, Muslim, age 25–34, Ethiopia]

*“During exam time, I assist her in her study at home during nights. I tell her to go to school and come back on time and to support her mother in domestic work. She usually studies her books at night. She is happy when I fulfill her needs like when she asked me to go to school.* ***”*** **[Male decision maker for girl able to cancel/postpone marriage, Merchant, Muslim, age ≥50, Ethiopia]**

*Program Support*. Girls’ and parents’ focus on completion of secondary education was facilitated by programs preventing early marriage. ODA, the program in Ethiopia, was frequently mentioned in this regard, as teachers were the program facilitators. While RISHTA, the program in India, was mentioned less often in these terms, when it was mentioned, it related to improving girls’ desire for education as a means of self-actualization.

*“Female teachers were convincing the girl’s family to let their daughter learn, mentioning how well the girl is doing in her education.”* **[Male decision maker for girl able to cancel/postpone marriage, Teacher, Orthodox, age 25–34, Ethiopia]**

*“As I heard about the proposal, I talked to her father. I told him that she was very promising student at our school level that she will be very important not only for her family but also for the country if she finishes he education.”* **[Male decision maker for girl able to cancel/postpone marriage, Teacher, Orthodox, age 25–34, Ethiopia]**

*“Firs, [ODA] helped me not to marry early. It also enabled me to continue my education. It helped me to convince my parents to postpone the marriage.”* **[Girl able to cancel/postpone marriage, Student, Muslim, age 13–17, Ethiopia]**

*“Initially, I wanted to complete my matric and then get married, but I joined RISHTA when I was in 10th standard. My life completely changed after that. Now, I wish to do something in my life. I want to study more and support my family financially. Besides, I also want to help other girls in the community to study more.”* **[Girl able to cancel/postpone marriage, Muslim, age 18–24, India]**

*Prospective In-Law and Groom Acquiescence*. Importantly, once girls and parents agreed to delay or postpone early marriage to keep girls in school, prospective in-laws and grooms often followed suit.

*“I was the one who talked about the possibility of postponing the marriage. I know that it was not the right age to receive grass [make promise]. I presented this fact in detail and her right to complete her education. The elders and boy’s parents accepted my idea and promised to wait until she complete grade ten. They said that they came to secure the promise, not to arrange marriage.”* **[Male decision maker for girl able to cancel/postpone marriage, Merchant, Muslim, age ≥50, Ethiopia]**

*“The girl’s family knew the proposing boy. And they say because he is educated (completed grade twelve), he is one of the guys who can support her with her education, and he is the one who works as a teacher in their village. However, they are sure that she has not reached at the right age for marriage, and they think that she has to complete her education before the marriage. The girl has currently been on continuing her education... The boy’s family have accepted the idea and thus reached on the agreement that the boy will continue to wait until the girl completes her education.”* **[Male decision maker for girl able to cancel/postpone marriage, Teacher, Orthodox, age 25–34, Ethiopia]**

##### Barriers to Continuation/Completion of Girls’ Secondary Education

Several barriers were indicated in terms of continuation of education in adolescence, including economic barriers such as financial costs of education, social barriers including social norms/expectations of adolescent girls and safety in mobility to attend school, and poor support or incentive for girls less academically inclined or interested to stay in school and delay marriage.

*Economic Barriers*. A primary barrier to girls’ continuation in school was economic in nature, with school fees viewed as burdensome, even if education was valued. Death of a father increased girls’ economic vulnerability to dropping out of school.

*Every girl goes to study these days. The government has launched so many schemes (e.g., incentive programs such as cash transfer programs) for this but there is still a lot of financial pressure in sending children to school. We find it difficult to deal with the pressure.* **[Female decision maker for girl able to cancel/postpone marriage, Housewife, Hindu, age ≥50, India]**

*“I heard a rumor from family members and my brother came to me told me I am going to get married… My brother told me, and I refused to accept the marriage. He said he can’t afford my educational expenses and said I should married to this man.”* **[Girl able** ***to*** **cancel/postpone marriage, Student, Muslim, age 13-17, Ethiopia]**

*“My father died when I was hardly 2½ years old. We use to work ‘mitti khodte’ [digging of earth], ‘dhan lagate’ [growing cereals] and earn our living. I could not study beyond 4th because of our poor finances.- I don’t have any wish to study further. Even if wanted to who would bear the additional expenditure?”* **[Girl married <18, Housewife, Sarna, age 18–24, India]**

Additional economic barriers to secondary education among poorer families in Ethiopia came from the need for girls to miss school in order to work in the markets selling items.

*“They (the girls) live on what they get from small business or on farming. For instance, today is a market day. They sell tea and other things to lead their life from what they get from such businesses. This causes them to be absent from their school.”* **[Male decision maker for girl able to cancel/postpone marriage, Teacher, Orthodox, age 25–34, Ethiopia]**

*“I understand is that due to shortage [of family funds], she is forced to miss class on market days to assist. She asked me for permission on Friday and Monday to go to market and engage in pity trading because she said she is not getting adequate assistance from her family. So, I support her and urge others to do the same in her education.”* **[Male decision maker for girl able to cancel/postpone marriage, Teacher, Protestant, age 18–24, Ethiopia]**

*Social Pressures*. Corresponding to community and family perceptions of potential disadvantage of education for marriage noted above, participants also described social pressures supporting discontinuation of education, including risk of social alienation for girls who continue their education and marry later. Again, these views were largely discussed in Ethiopia and not India.

*If a girl delays her marriage, she might be socially shunned and treated as older. Clever students who decide to continue with their education and postpone their marriage are most often mistreated in the community.* **[Male decision maker for girl able to cancel/postpone marriage <18, Teacher, Protestant, age 18–24, Ethiopia]**

*“Delaying marriage has no disadvantages in terms of science. But the society makes her suffer psychologically, because they think as no one wants to marry her. They always laugh at her in schools and in her locality. Thus, brilliant students because of this fear of social stigma, they dropped out of school.”* **[Male decision maker for girl able to cancel/postpone marriage, Teacher, Orthodox, age 25–34, Ethiopia]**

In part, girls’ continuation in school as adolescents was not valued due to lack of faith in girls’ capacity to learn and advance in school. There was also concern that knowledgeable girls would not adhere to appropriate behavioral norms or become unable to adjust to marriage and thus cause family shame.

*“They [girls] lack attention and equal treatment in this society. There are many girls who have the talent and skill… They think that girls can’t get higher… They still think of girls as inferior. The community and mothers influence them to get married. There are girls who are forced to marry without their consent.”* **[Male decision maker for girl able to cancel/postpone marriage, Teacher, Orthodox, age 25–34, Ethiopia]**

*“In our locality some people don't give due attention for grown up [adolescent] girls’ education… Those people say girls never succeed in education.”* **[Male decision maker for girl married <18, Farmer, Muslim, age 25–34, Ethiopia]**

*“The community doesn’t believe in educating girls. There is a proverb that follows in relation to this. For this reason they don’t want to send girls to school. If they do, they think that girls will learn some practices that will cause her parents to be ashamed.”* **[Female decision maker for girl able to cancel/postpone marriage, Teacher, Orthodox, age 18–24, Ethiopia]**

*“But there are people in the society who have a negative opinion about an educated girl. They say ‘She is an educated one, brother, and would not adjust easily [to marriage].’”* **[Girl able to cancel/postpone marriage, Teacher, Sarna, age 25–34, India]**

Too often, these social pressures resulted in girls being pressured into marriage and forced to leave school.

*“My wish was to continue my education. I got married because I don’t have the chance to refuse it is not because I think the marriage is right thing to do.”* **[Girl married <18, Student, Muslim, age 13–17, Ethiopia]**

*Risk for Harassment*. Girls in Ethiopia also faced harassment as they went to and from school, and noted this as a barrier to school continuation.

***“*** *The case is that young men attempted an assault and harassment on her while she was to school and she was psychologically hurt… We reported to court. There are two girls victims of the harassment.”* **[Male decision maker for girl able to cancel/postpone marriage, Merchant, Muslim, age ≥50, Ethiopia]**

*“They are persons used to abuse us just standing on the road while we go and back from school. They have no job to perform… They use to insult us… So, I stopped them from insulting me by telling my parents, my brother and my mother. And now they are no more passing on the way I use after recognizing my self-assurance.”* **[Girl able to cancel/postpone marriage, Student, Muslim, age 13–17, Ethiopia]**

*Lack of Support or Options for Girls Less Academically Inclined*. Support for ongoing education of girls who were higher performing and more motivated was well-documented in both country contexts. These girls were also the ones best able to continue education even after marriage.

*“The girl’s family ascertained that since she has been [motivated about]her education, her family will not allow her to marry and that decision will be firm until she completes her education, even in case she wanted to accept the proposal. Of course, the girl’s family knew how ambitious and how willful she has been on her education.”* **[Male decision maker for girl able to cancel/postpone marriage, Teacher, Orthodox, age 25–34, Ethiopia]**

*“She was always happy about her education. She was happy when she went to school and when she came back. She was happy when she studied too. She was cleverest of all my children... When I heard she started her education at her husband’s place, I became very happy. Her father wanted her to be educated. But, she married on her own will.”* **[Female decision-maker for girl married <18, Farmer, Muslim, age 35–49, Ethiopia]**

However, for the girls less inclined and interested in education, leaving school to marry was welcome. Additionally, parents were less focused on supporting these girls to continue their education.

*“I didn’t actually experience challenging life even once. I stopped education willingly and I married willingly too. Nobody forced me to quit education. So I didn’t face any problem… Whoever wants education can learn and others can marry.”* **[Girl married <18, Housewife, Muslim, age 18–24, Ethiopia]**

*“I wanted her to study and send her school but she was not a good student. And how would she pass her exam and get promoted to the next class. My elder daughter was good at studies so we let her complete her education and refused the proposals which used to come for her. But later after she turned 20 proposals stopped coming for her. In case of (my younger daughter), she was not good in studies and I could see that she had lately started befriending boys. I was always scared that she may get involved in an affair with some boy of other cast and run away with him. We wanted her to study more but she was becoming friendly with boys. I didn’t like it and so as soon as we received a good proposal we fixed her marriage. Now she is happy and blessed with a baby boy.”* **[Male decision-maker for girl married <18, Agricultural worker, Sarna, age 35–49, India]**

*“My childhood time is from age 10 to 16, which I spent by playing with friends but after I became 17 years old I perceived myself as an adult. As the man married me and my parents discuss about the marriage I married. This is because while attending school I was so sick many times. As I was frequently face headache. I did not attend education well. I thought I am not attending education well and I planned to get married. Accordingly, I got married.”* **[Girl married <18, Student, Muslim, age 18–24, Ethiopia]**

#### THEME 3. Enablers and Barriers to Education Post-Marriage

##### Enablers of Continuation of Girls’ Education Subsequent to Marriage

**Fig. 3:**
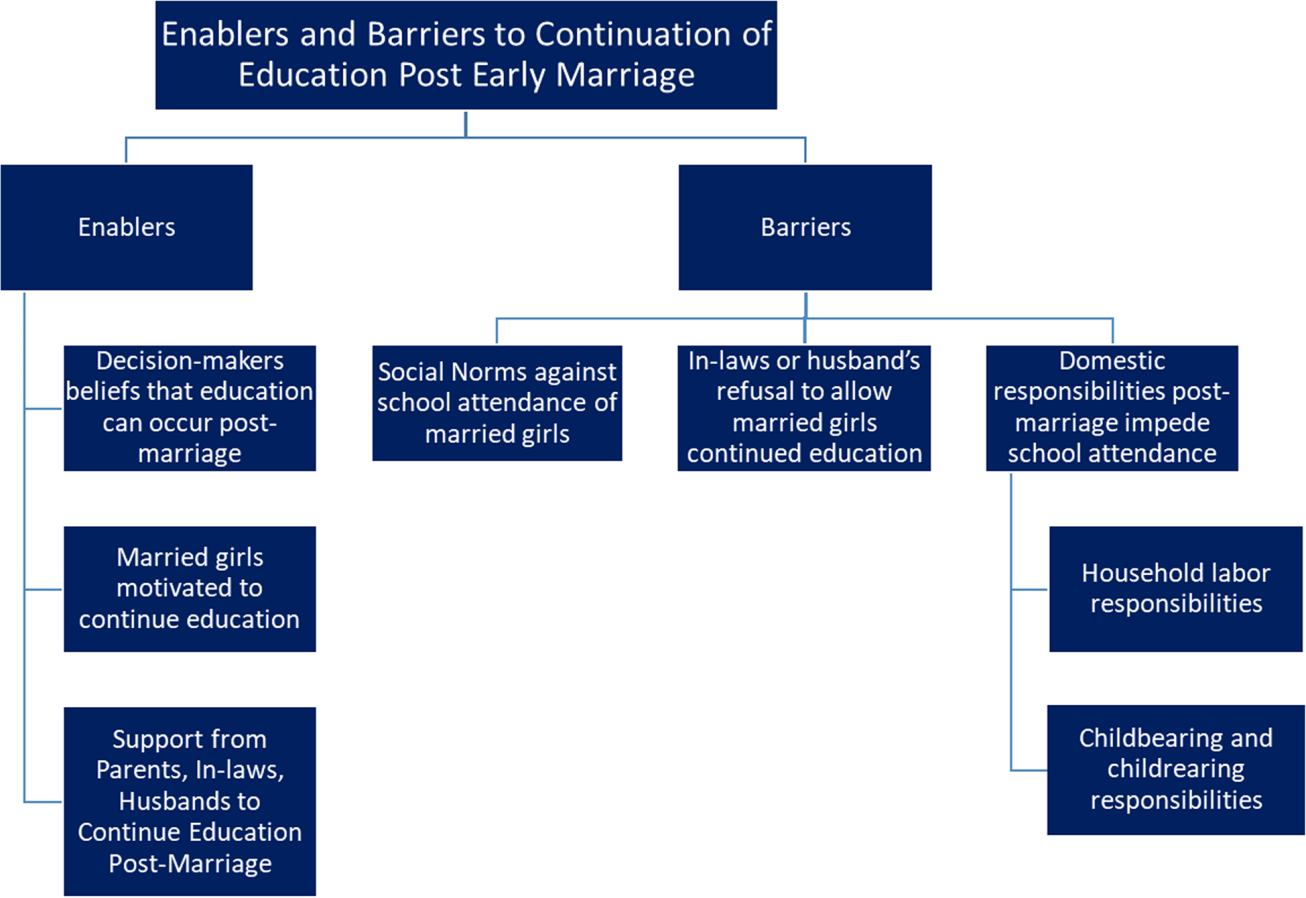
Enablers and Barriers to Continuation of Girls’ Education Post-Marriage: *Note: Blue indicates findings reflect Ethiopia and India*

While post-marriage education was not the norm, it was discussed by some participants. Key enablers were perception of social acceptability for girls, girls’ motivation to continue education post-marriage, and support for continued education subsequent to marriage from her family as well as her husband and in-laws (Fig. 3).

*Decision-Makers’ Beliefs that Post-Marriage Education is Possible*. Given the context of low girl education and high marriage-related dropout, an important enabler was belief that ongoing education after marriage or birth is an option. Often, decision-makers reported this belief, particularly in Ethiopia.

*“It is good to continue with education after marriage if they can do that. She has right to continue with her education even after giving birth. She can go on her education while managing her home.”* **[Female decision maker for girl married <18, Farmer, Muslim, age 25–34, Ethiopia]**

*“A married girls has to go to school because marriage should not affect her education. Nothing can harm her even if she completes after marriage*.” **[Female decision maker for girl able to cancel/postpone marriage, HEW, Muslim, age 18–24, Ethiopia]**

*“I believe a married girl should stay in school. If it is possible she should not marry, if not she should continue her education being married, for example, the one who is going to marry her and her parents can prepare an oath that he will make her continue her education*.” **[Male decision maker for girl able to cancel/postpone marriage, Teacher, Orthodox, age 25–34, Ethiopia]**

*“A girl should continue her education as far as her choice. She can and she must complete her education. The husband should support her so that she can also study after her marriage. Why should marriage be a limiting factor?”* **[Male decision-maker for girl able to cancel/postpone marriage, Health Worker & Farmer, Hindu, age 25–34, India]**

*Married Girls Motivated to Continue Education Post-Marriage*. In India, there was much discussion regarding the importance of the girl’s motivation to pursue education post-marriage, as it is more normative in that context.

*“Yes, she can study after marriage also. It all depends on her and her convincing power. There would be additional responsibilities but if she wants she can manage the affairs accordingly.”* **[Girl able to cancel/postpone marriage, Student, Hindu, age 18–24, India]**

*“Girls can study till any level, but they should at least study till intermediate, as these days anything less than that don’t really serves the purpose. If she gets married early before completing her intermediate, she may study after marriage also, but it depends on her concentration.”* **[Girl able to cancel/postpone marriage, Student/Housewife, Hindu, age 18–24, India]**

*“The girls can stay in school so long as they like to study. I would not want to restrict my wife in studying.”* **[Male decision-maker for girl married <18, Farmer, Sarna, age 18–24, India]**

*Support from Family, In-Laws and Husbands to Continue Education Post-Marriage*. In both contexts, girls reported that ongoing education requires a broad range of support, from both natal and in-law family, as well as husbands and even schools. Often, parents and girls selected grooms based on their promise to support her continued education.

*“My family including my husband and his parents are now supporting me. They want me continue with my education and improve my life and theirs. My teachers have also been supporting me.”* **[Girl married <18, Housewife, Muslim, age 18–24, Ethiopia]**

*“As the proposed groom (my husband) is also an educated person, his friend has told me that I would not be harmed and I would not drop out school though I married… As both my parents and the proposed groom are interested to continue my education I accepted the proposal*.” **[Girl married <18, Student, Muslim, age 18–24, Ethiopia]**

*“I was happy if she could manage to learn at least up to grade 5, be it under the supervision of her husband or her parents. He promised to help her learn till she complete her education… We let him marry. He promised to facilitate and help her complete her education. But the engagement is already done.”* **[Male decision maker for girl married <18, Farmer, Muslim, age 25–34, Ethiopia]**

*“My parents found me a good match, as he is 27 years old, has a business of his own and is from a good family. His father is not alive and he is the head of the family. He is intelligent and has promised that he will let me continue my education after marriage.”* **[Girl married <18, Sahara Agent, Hindu, age 13–17, India]**

##### Barriers to Continuation of Girls’ Education Subsequent to Marriage

Social norms restricting education for married girls, restrictions from in-laws and husbands against continued education following marriage, and responsibilities of married life and motherhood complicated continued education of girls, even for highly motivated girls.

*Social Norms against School Attendance for Married Girls*. In both contexts, social norms dictated that school was accessible only for unmarried girls, though this was more explicitly described in Ethiopia.

*“Yes, if she goes to school, there is no problem that she may face. However, in practice what we see in the society is a married boy going to school not a married girl*.” **[Male decision maker for girl able to cancel/postpone marriage, Teacher, Orthodox, age 25–34, Ethiopia]**

*“It is better if they complete their education. It is common that education is impossible after marriage. They must complete their education before marriage because it is easy for them to manage before marriage.”* **[Girl married <18, Student, Muslim, age 18–24, Ethiopia]**

In India, more restrictive language was used, with participants directly saying they did not approve of married girls going to school; one participant even erroneously alleged that school attendance of married girls as illegal.

*“According to me studying after marriage is not a good idea.”* **[Girl married <18, Housewife, Hindu, age 13–17, India]**

*“A married girl stay should not be allowed to go to school*.” **[Female decision-maker for girl married <18, Agriculture, Muslim, age ≥50, India]**

*“No, a married girl cannot continue her education after her marriage as it is not permissible by our law to allow a married girl go to such places after marriage*.” **[Female decision maker for girl able to cancel/postpone marriage, Shop Owner, Muslim, age 35–49, India]**.

*In-Laws or Husbands Refuse Girls’ Continued Education*. In both contexts, in-laws and husbands were the decision-makers regarding education continuation. They were often unsupportive, particularly in-laws.

*“His family is against me. They are complaining about my education…. They also want [me] to give birth soon. They say that giving birth better*.” **[Girl married <18, Student, Muslim, age 18–24, Ethiopia]**

*“My husband and parents want me to study further, but my in-laws are not interested to continue my studies. Moreover, what will I do with higher studies, as ultimately I have to take care of my family*?” **[Girl married <18, Housewife, Sarna, age 18–24, India]**

*“If you ask me, I will say definitely, ‘Yes, she should continue.’ But I don’t think anyone in our community will allow their daughter-in-law to continue her education.”* **[Girl married <18, Housewife, Muslim, age 13–17, India]**

*“A married girl can go to school only the in-laws are liberal enough. Otherwise, there will be tension and fights at home. It's better to have a common decision. Usually, the in-laws would decline the idea of married girls attending school.”* **[Male decision-maker for girl married <18, Farmer, Sarna, age 18–24, India]**

There were also instances described in which husbands or in-laws agreed to support girls’ continued education post-marriage at the time of engagement, but refused support once the marriage occurred.

*“The person who intended to marry her promised to let her continue her education after marriage, and her family decided to proceed with her marriage… but after her marriage she couldn’t continue her education… He did not allow her to learn and did not kept his promise and she became very sad.”* **[Male decision-maker for girl married <18, Farmer, Muslim, age 25–34]**

*Domestic Responsibilities Post-Marriage Restrict Education Access*. Both decision-makers and girls across country contexts reported that, even if there was girl interest and in-law and husband support for her ongoing education, domestic responsibilities as a wife restricted this as an option.

*“It’s difficult to study after marriage, as it is there are so many new responsibilities and roles that girl has to play after marriage in her new home. On top of that if you burden her with study then it is very difficult for the girl to cope.”* **[Male decision maker for girl able to cancel/postpone marriage, Agriculture, Hindu, age ≥50, India]**

*“Including married girl, all should go school if possible for no limit to learn and know more. But there are few in numbers who go back to school after marriage due to responsibilities they are experiencing at home*.” **[Female decision maker for girl able to cancel/postpone marriage, Teacher, Protestant, age 25–34, Ethiopia]**

*“After marriage, it has importance for the married girl to stay in school. Some start learning until they become pregnant, after this they stop. In addition, since most are farmers, they have to prepare food for their husbands and help them in farming; this condition does not allow them to continue their education after marriage*.” **[Female decision maker for girl able to cancel/postpone marriage, Teacher, Muslim, age 25–34, Ethiopia]**

Domestic burdens as a restriction to ongoing education of girls are exacerbated by motherhood.

*“She should complete her education before having her first baby. Because it is very difficult to continue with studies after having a child*.” **[Girl able to cancel/postpone marriage, Student, Sarna, age 18–24, India]**

*“After we had child, who is responsible to care for the baby, who manages the house, it is not husband but it is the wife… There are many challenges after marriage, managing the house, caring for the child. As girls we have lots of responsibilities, so I think that they should finish their education before they marry*.” **[Female decision maker for girl able to cancel/postpone marriage, Teacher, Orthodox, age 25–34, Ethiopia]**

*“Well, once they marry, it is inevitable for them to have baby. If they delay to have baby for the time being, it is a must for them to have baby in the future. When that happens, it might have effect on their education* ***.”*** **[Male decision maker for girl able to cancel/postpone marriage, Teacher, Orthodox, age 25–34, Ethiopia]**

Even in cases where there was spousal support for ongoing education post-marriage, maternal responsibilities often rendered educational opportunity impossible for married girls.

*“I insisted many times with him (my husband) to wait until I was 18 to get married, but he did not agree. Finally we ran way in some 7 months from a mela. After marriage also, he loves me and respects me. He got me admitted to 9th standard. I even went to school for 5 months after which I got pregnant and was not able to continue my education.”* **[Girl married <18, Housewife, Sarna, age 18–24, India]**

## Discussion

The present study builds on previous quantitative work documenting the importance of education retention to prevent early marriage [20, 22, 40] by qualitatively exploring the beliefs, norms, and practices of girls’ education and their relation to early marriage, as described by girls exposed to early marriage prevention efforts in rural Ethiopia and India, as well as these girls’ marital decision-makers. Our analysis highlights the risk context impeding girls’ education, but also resiliency factors that support girls continued education in both of these settings.

### Risk Contexts for Girls’ Education

Corresponding with quantitative data from these regions documenting high rates of early marriage and poor levels of girl education [18, 19], and prior research demonstrating that girls’ education is often devalued in contexts where early marriage is supported [10, 12, 14, 24], social norms against education of girls were heavily noted by communities. These norms are further reinforced by traditional gender norms in the community, such as beliefs that girls cannot learn as effectively as boys and that educated girls who delay marriage are more likely to be defiant to parents and resistant to marriage. Further, many indicated fear that girls who continued education and delayed marriage were more likely to date or engage in premarital sex, which would bring dishonor to the family and community. Such norms affect girls’ treatment and support in education but also their value on the marriage market, with many indicating that educated and older girls were less likely to be selected for marriage or may have fewer options for a good match. Unmarried educated girls can also be shunned by their communities, a finding in line with prior research [35, 54–56]. In sum, traditional gender norms devalued girls’ education and educated girls themselves, and reinforces girls’ value based on her marriageability, thus creating a context less supportive of girls’ completion of secondary education. These persistent traditional norms impede gender development [57–59], including inadequate progress on women’s education levels, employment and career advancement [18–20].

Beyond social norms restricting education in these contexts, certain structural factors also compromised girls’ school attendance and completion. In Ethiopia more than India, respondents highlighted the financial costs of education as an impediment, particularly for the poorest girls. As seen in prior research [22], school fees, low attendance due to income generation needs, and inadequate employment opportunities (particularly for girls) offer an uncertain economic cost-benefit ratio. Additionally in Ethiopia, but not India, many girls and decision-makers described abuse and harassment of girls in public spaces as they went to and from school. Poor infrastructure (e.g., no roads), and permissiveness regarding public harassment of girls [60], contribute to this problem. An additional concern identified in these analyses, in both India and Ethiopia, is for girls who are less academically inclined and motivated. Efforts in school retention are built upon an assumption that girls want to stay in school, but this is not always the case. Low resource families may be understandably disinclined to force education on girls in the above described contexts where such force is going against local norms and may compromise marital prospects, when marriage is often viewed as the best option and opportunity for economic security for girls.

Subsequent to marriage, school continuation opportunities were even more constrained by social norms and policies. Additionally, education decisions were more typically not under girls’ control once married, but instead controlled by in-laws or husbands who were often unsupportive of ongoing school attendance (sometimes irrespective of what had been agreed up on prior to marriage). For married girls who were supported to continue their education, domestic responsibilities, particularly those related to child rearing, became impossible barriers to overcome. These findings are consistent with prior research from even high resource settings documenting that domestic responsibilities post marriage and childbirth detract from continued education.

### Resiliency Factors for Girls’ Education

Girls and decision-makers also described a number of psychological, social and structural assets and resources that supported girls’ retention in schools, in line with resiliency theory [45].

#### Girls’ and Decision-Makers’ Awareness of Benefits of Girls’ Education

Despite the many constraints against continued education of girls that instead push them into early marriage, benefits of education were well-recognized by participants. Decision makers in particular recognized that educated girls can have greater capacity, particularly through literacy, to navigate society and build life skills. In India, improved domestic capacities, including child rearing, were also an appreciated benefit of girls’ education, where in Ethiopia, improved financial capacity to manage household resources was recognized. Importantly, despite norms of non-employment of women in these contexts, many participants recognized the importance of education to enable employment and financial security for women. Girls in particular discussed the value of education for economic self-sufficiency, including career opportunities and financial independence. In India, this more often focused on stereotypically female career aspirations (e.g., seamstress, teacher, etc.) and for the financial benefit of their family, rather than as a personal benefit; where in Ethiopia, the potential benefits of job attainment and income generation were linked to the ability to be an autonomous agent without need for a husband. The latter suggests a broader norm shift and greater emphasis on economic empowerment in Ethiopia, possibly because the program was conducted by teachers and eventually included an economic component in the form of a savings plan for girls [49]. Similarly, emphasis on traditionally female cottage industries for employment among girls in India was possibly due to focus on skills building in these areas in the Indian intervention [50]. Importantly, in both country contexts, respondents, girls and decision-makers, recognized the value of girls’ education to support economic security and development of communities and families.

#### Girls Educational Self-Efficacy and Social Support from Family and Community Programs

Fundamental to girls’ continued education in these contexts are the cognitive strengths of the girls themselves; those with the capacity and motivation to excel academically, combined with the self-efficacy to voice their preferences, had higher school retention. Girls who voiced their desire for education were better able to generate and receive social support from their parents and teachers. In Ethiopia, teachers were particularly highlighted as an important social support to help girls stay in school, primarily by encouraging girls’ parents to see the value of education. The role of teachers in Ethiopia may have been more pronounced as the delayed marriage intervention in that context was based in schools and delivered by teachers. These teachers discussed the importance of staying in school in part as a means of deterring early marriage, and as a human right for girls. In both India and Ethiopia, programs were identified as being important social supports to girls for education and delay of early marriage, and for girls’ self-efficacy in raising their voice to parents on these issues. Importantly, once girls and parents were in agreement on these issues, they were better able to gain support for delayed or postponed marriage and continued education with prospective in-laws and grooms. These findings are consistent with prior findings from this research regarding the importance of girls’ use of their voice and parents support for girls’ decision-making resulting in girl-led education retention and delay of marriage [61].

#### Married Girls’ Educational Self-Efficacy and Family Support for Girl Education Post-Marriage

Corresponding with increased barriers to education subsequent relative to prior to marriage, few participants recognized enablers and facilitators for school retention of married girls. Those that did highlighted key assets and resources seen for retention in school prior to marriage: self-efficacy and motivation of the girl combined with family support. However, for married girls, family support required inclusion of in-laws and husband, as well as the natal family. An additional necessary aspect was decision-makers’ beliefs that continued education is possible subsequent to marriage; these beliefs may be particularly important for married girls, given the stronger social norms against continuation of education for girls once married.

### Financial Assets and Policy Considerations

While financial barriers were noted as an impediment to girl education, and prior research indicates that cash transfer programs show promise in retaining girls in school and delaying marriage [20, 62, 63], findings from the current study did not directly identify financial assets as necessary to keep girls in school. Both Ethiopia and India have active efforts to increase girls’ secondary school retention through incentivization schemes. In Ethiopia, programs like Berhane Hewan, established in 2004, offer economic incentives for enrolling girls in school, including school fees, livestock, school materials, and eventually a financial literacy curriculum [40]. At the Woreda level, Kebele (neighborhood) Education Training Boards provide meal programs at school and cash transfers to parents who have girls attend school [64]. In India, the government has a mid-day meal requirement at school [65], as well as a number of educational incentive programs, including Apni Beti Apna Dhun (“Our daughter, our wealth”)- established in Haryana in 1994 to provide cash transfers for parents whose girls graduate secondary school [66], the Cycle Program- established in 2006 in Bihar to provide bicycles for girls attending school [67], and the National Scheme of Incentive to Girls for Secondary Education- a national cash transfer program launched in 2008 [68]. Despite these efforts, inadequate progress has been made, and gender equality in educational attainment has yet to be achieved [18, 19]. This reinforces current findings, which suggest that efforts toward norm change, building empowerment and resiliency of girls, increasing options and value for girls beyond marriage, and providing social support for girls to pursue these options are all needed. The benefits of such an approach can be substantial, given the already proven value of education retention to delay early marriage [20–22] and the social and health benefits of women’s involvement in household finances and financial decision-making [31–33, 69–71]. Alternatively, if we remain in the current trajectory, the value of girls’ education will remain minimal, and that education may even compromise their marital options and safety, as has been seen in some settings [72].

### Limitations

While the current study offers important insights into how perceived benefits and costs of girls’ education affects early marriage, results must be interpreted in light of study limitations. The study employed purposive sampling in the Oromia Region, Ethiopia, and Jharkhand State, India, with and through girls exposed to early marriage prevention programs, and thus results may not be generalizable beyond this sample. Analyses that directly linked girls and decision-makers were not feasible due to data limitations. Data may reflect some social desirability bias, as respondents were exposed to programs focused on early marriage and the value of school retention, though this may be mitigated by the fact that most respondents were no longer in the programs at the time of interview. Program implementation was slightly different in both regions; data from Ethiopia were drawn from girls in school, in a context of low school enrollment, whereas data from India were drawn from a community-based sample. While 58 interviews were dropped from the analysis due to data quality concerns, a total of 207 interviews were retained and analyzed. Further, self-reported interviews conducted with an interviewer are subject to reporting bias, particularly when discussing sensitive subject matter such as early marriage. In order to decrease reporting bias, interviews were conducted in private rooms and interviewers external to ODA and Project RISHTA were hired to interviews participants. Finally, study findings are limited by the fact that original data collection was not designed to focus on girl education outside of its relationship to early marriage, therefore we were unable to deeply explore issues commonly related to dropping out of school (e.g., long travel distances). Nevertheless, the current study provides a unique lens into the value, barriers and enablers of education of girls in contexts with a high prevalence of early marriage, providing important insights for program and policy improvements.

### Conclusion

Findings from this qualitative study of girls who participated in early marriage prevention programs and their marital decision-makers in the context of Oromia, Ethiopa and Jharkhand, India, regions with high rates of early marriage of girls, demonstrate recognition of the value of girls’ education and subsequent delayed marriage in terms of improved opportunity to build life skills, stronger domestic capacities to manage a household and care for children, and greater capacities to manage household finances or pursue career opportunities. Nonetheless, participants also recognized that social norms that discourage girls’ education and encourage early marriage persist, and these can result in backlash in the form of stigmatization and social alienation of those who do not adhere to these norms. Such norms, combined with norms against women’s employment and inadequate career opportunity for women through education, directly impede the value of education in these regions. Nonetheless, girls’ internal motivation, academic capacities and self-efficacy can overcome these barriers, particularly when supported by family and programs, particularly programs that engage teachers. Our results suggest that while promotion of girls’ education has good social value for many in these contexts, and can reduce early marriage, such approaches will be inadequate in the absence of social norm shifts and structural changes toward women’s autonomy and broader career opportunities. These findings speak to the value of a strengths and resiliency-based response to supporting early marriage prevention, by building assets and capacities for girls, and an enabling environment in which to apply them. Educating girls in the absence of broader social change to build upon girls’ developed strengths and with a gender equality lens will be inadequate to address early marriage and the sequelae of social and health concerns that accompany it.

## Additional file

Additional file 1: Interview Guides. (PDF 590 kb)

## Abbreviations

ODA: Oromia Development Association
Project RISHTA: Regional Initiative for Sexual Health for Today’s Adolescents
