# Supplementary material for: Students and brides: a qualitative analysis of the relationship between girls’ education and early marriage in Ethiopia and India
Source: BMC Public Health. 2019 Jan 7;19:19. doi: 10.1186/s12889-018-6340-6 (PMC6322316; doi:10.1186/s12889-018-6340-6)
Supplement: Supplementary file 1 — Interview Guides. (PDF 590 kb) [file 12889_2018_6340_MOESM1_ESM.pdf]

Interview Guides: Ethiopia

In-Depth Interview

1. Participating girls under age 18 whose marriages were cancelled or postponed;

Step 1: Obtain permission from a parent or legal guardian for the participation of girls under age 14.
Step 2: Obtain consent from all respondents before beginning the interview.
Step 3: Begin the interview

Socio-demographic

Region______________Zone____________Woreda____________School/kebele___________
Age____ Educational level_________ Religion________________
HH owner____________ Are you_____ child in your family. Family income source___________
Date of the interview____________ Start time __________end time _____________
Name of Interviewee____________________
Responsibility ________________________ Supervisor_________________

PART 1: KNOWLEDGE, ATTITUDE AND PERCEPTION ON EARLY MARRIAGE AND FIRST BIRTH

First I would like to talk with you about your opinions about the age when girls should marry, and what you see as the benefits and disadvantages of early marriage.

1. What does it mean to be a grown up as an adolescent in your locality? Can you tell me about your growing-up from childhood to date? When was the time you enjoyed the most? Did you have times that were really challenging? (probe until you get ample background information)

2. At what age do you think girls should get married? What are the benefits and disadvantages of delaying marriage until at least age 18? Can you tell me the social, economic, health and educational benefits or disadvantages of delaying marriage?

3. At what age you think girls should have their first baby? Can you tell me what you know about early childbirth?
   a. How would it affect someone like you to have a baby before age 18?
   b. Where did you get this kind of information? Did you get information from clubs? What about from life skill training? From other ODA activities? Any other sources?

4. What do you think about girl's education? Do you think girls should go to school like boys do? What will happen if you don't go to school?

5. Up to what level should a girl continue her education before getting married? Why?

6. What is the highest level of education you would like to accomplish? And what kind of profession/job/ would you like to engage in? Up to what level should a girl continue her education before having her first baby? Why?

PART 2: CAUSES AND CONSEQUENCES OF EARLY MARRIAGE AND FIRST BIRTH

Next I would like to talk with you about the causes and consequences of early marriage and first birth now you are an adolescent.

1. At what age do most girls get married in your community? What are the reasons that girls get married before the age of 18?

2. What did you think about the information you received on the benefits of waiting until age 18 to marry? Were you satisfied or dissatisfied? Why?

3. What did other people think about you seeking information? Were they supportive? Was anyone against it? Why?
   - What about your immediate family members? What do they think? What do your brothers and sisters think? What about your friends?

Now I would like to discuss using the following mapping exercise:

SOCIAL NETWORK (Mapping)

Note to interviewer: *You will use 2 pieces of paper with a "map" of concentric circles on each (see example below). The smallest, center circle will be marked as "I". Subsequent circles will be marked as family, peers, community members including health workers, teachers and people outside the community. Using the questions below you will ask the respondent to reflect upon the people who provide them with informational support (such as helping or advising) related to early marriage and first birth, and individuals who pose barriers. You or the respondent will*

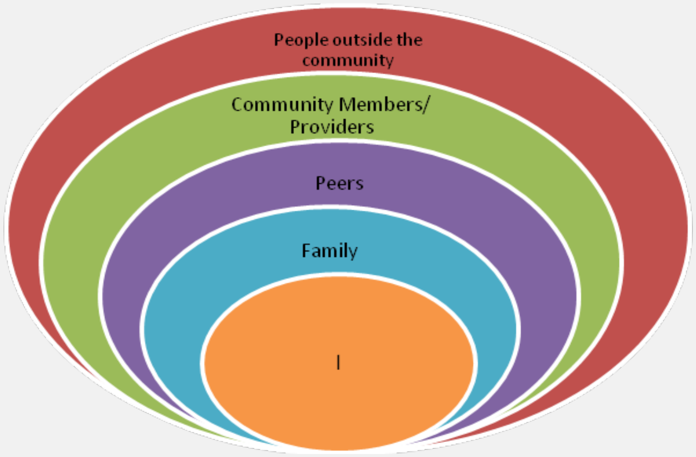

write each of these people into the appropriate circle.

I would now like to talk with you about whom you talk to and how you get information related to the canceling/postponement of your marriage. Let's start at the smallest circle. This circle is you. The other circles represent your family, peers, community members and health providers, teachers and people outside the community.

1. Now let's talk about people who support you by sharing information about early marriage and first birth, such as advising you on what to do?
   - Who in your family provides this support?
   - Peers?
   - Other people in the community? What about HEWs and health workers? Teachers?
   - People outside of the community?

2. Is there anywhere else you get information about delaying marriage and first birth or how to stay unmarried? For example: Radio or television?

3. Now let's think about people who may act as **barriers** or present obstacles related to canceling/postponing your marriage:
   - Who in your family?
   - Peers?
   - Other people in the community? What about health workers? Agricultural workers?
   - People outside of the community?

4. Lastly, I would just like to ask you a few additional questions about these people and sources of information.
   a. Which of these people or other sources do you trust the most? Why?
   b. Which of these people or other sources do you trust the least? Why?
   c. If you wanted to delay marriage and first birth whose support would be most important to you? What are the reasons for this answer?

PART 3: DECISION MAKING PROCESS

I would like now to talk about the decision to canceling/postpone your marriage.

1. First, how was the marriage proposal made, and by whom? And why was this individual propose?
   - Was the marriage proposal presented to you or did you hear about it through somebody else?
   - If the proposal directly came to you, who told you about first?
   - What was the age of the boy/man who proposed marriage to you?

2. Who first talked about the possibility of cancelling/postponing your marriage? Then what happened? Please briefly tell me the chain of events that eventually led to the marriage being cancelled/postponed.

3. Who made the final decision? IF IT WAS NOT THE GIRLS, ASK:

- How did you learn of their decision?
- Do you usually discuss marriage and girls education within your family? Did you discuss cancelation and postponement of the marriage? Was there agreement among all family members about the decision? If postponed, do you still have a relationship with this person? If yes, how do you describe your relationship now? Do have any sexual relationship? Did you use any protection or family planning method? Where did you get the family planning method?
- How easy or difficult were all of these decisions? Why?

4. How did the decision maker(s) come to their decisions, and what role, if any, did the ODA project play in these decisions?

5. Were there other people who were influential in the decision? If so, who? How did they influence the decision?

6. IF THE GIRLS DECIDED, ASK: Who supported you in your decision process, and in what ways?

7. What support did he/she/ they have given you?

8. How easy or difficult was the decision making?

9. Who in your family usually has the final say on marriage decisions?

10. Do you still think the decision was the right one?

PART 4: ENABLING FACTORS

1. What were the factors that made it possible for you and your family to cancel and/or postpone the marriage?

2. What do you know about existing laws and regulations about early marriage?
    - How do laws and regulations affect someone like you?
    - How did you learn about the existing laws and regulations on early marriage? Did ODA have something to do with it?

3. Can you tell me any of the school factors (Gender responsiveness), such as teachers' support for canceling/postponing your marriage?

4. Can you tell me about community support you received for the decision to cancel/postpone your marriage? For example, how supportive other families were, law enforcement bodies, health service workers, others?

5. How did your proposed groom react to the cancellation/postponement? How did his family react?

6. What was your experience with ODA interventions (sport for life, community awareness, and award provided to parents, saving for schooling, counseling, referral, girls club etc).
   - Did these activities help you to cancel/postpone your marriage? How?

7. If not in a relationship with the proposed groom, ASK: Are you in romantic relationship with someone else? Are you involved in a sexual relationship with this person? Do you use any contraceptive? How did you come to know about contraceptives? Where do you get the contraceptives?

Then I would like to talk with the people who made the decision to cancel/postpone your marriage. I know this was a big decision for your family, and we are interested in hearing from these people how they came to make this decision.

1. Of all the people who supported you in the canceling/postponement of the marriage, who do you think are the most important ones? Can you tell me the one, two or three people you think that, without their support, things would have ended differently? You don't have to name three people if only one person or two made the decision.
2. I really want to speak to them, do you mind?

Now I would like to discuss using the following mapping exercise:

PART 5: PERCEPTION OF ODA ACTIVITIES (Free-listing)

In the last part of this interview, I would like to discuss your thoughts about ODA activities;

1. First, let's think about the ODA activities in your community regarding early marriage and first birth;
   a) Can you describe what role ODA plays in the community in delaying marriage and first birth?
   b) Can you tell me three words that would come to your mind if you were asked to describe ODA activities regarding girls' attitudes toward gender equality, early marriage and their perceived role in deciding when to marry?
   c) Next, can you tell me three qualities that come to your mind about ODA's activities?

2. How do you think your participation in ODA activities did affected your relationships with their parents? What did you benefit from ODA activities help you to improve your relationship with your parents?

3. Now, let's think about ODA's project to change the relationship between the girls and their parents.

   a) Can you describe the role that ODA plays in changing the relationship between girls and parents in the community? If so, how? Were there challenges along the way?
   b) Can you tell me three words that would come to your mind if you were asked to describe ODA's activities regarding changing the relationship between girls and

parents (Note: for example, is there any meaningful discussion between girls and parents?).

c) Next, can you tell me three qualities that come to your mind about ODA's activities in changing the relationship between girls and parents?

PART 6: PERCEPTION OF SERVICE PROVIDERS (Free-listing)

In the last part of this interview, I would like to discuss your thoughts about local health workers and school teachers.

1. First, let's think about your local health workers or health extension worker.

    a) Can you describe what role these workers play in ODA interventions on early marriage and first birth?
    b) Can you tell me three words that would come to your mind if you were asked to describe the service that they have provided to you through their youth friendly health services? (Note: if no care from youth friendly health services, ask about general care on sexual and reproductive health (SRH)).
    c) Next, can you tell me three qualities that come to your mind about this provider?

2. Now, let's think about your local school teacher.

    a) Can you describe what role these workers play in ODA interventions on early marriage and first birth?
    b) Can you tell me three words that come to your mind if you were asked to describe this worker regarding the SRH and youth friendly services they have provided (Note: for example, at which age it is appropriate to get married).
    c) Next, can you tell me three qualities that come to your mind about this provider?

CLOSING

Thank you very much for your time and for answering these questions. Before we end this interview, is there anything else you think we should discuss?

Thank you. I would like to assure you again that everything we have discussed today will be kept confidential.

In-Depth Interview
2. Participating girls who were married before age 18

Step 1: Obtain consent from all respondents before beginning the interview. Married girls do not need permission from parents or guardians to participate.
Step 2: Begin the interview.

Socio-demographic

Region______________Zone____________Woreda____________school/kebele___________
Age____ Educational level_________ Religion________________
HH owner____________ Are you_____ child in your family. Family income source___________
Date of the interview____________ Start time __________end time _____________
Name of Interviewee____________________
Responsibility ________________________ Supervisor_________________

PART 1: KNOWLEDGE, ATTITUDE AND PERCEPTION ON EARLY MARRIAGE AND FIRST BIRTH

First I would like to talk with you about your opinions about what you see as the benefits and disadvantages of marrying before age 18, and having a baby right away.

1. What does it mean to be a grown up as an adolescent in your locality? Can you tell me about your growing-up from childhood to date? When was the time you enjoyed the most? Did you have times that were really challenging? (Probe until you get ample background information).
2. At what age you think girls should marry? At what age should they have their first baby? Can you tell me what you know about early marriage and first birth?
   a. How does it affect someone like you?
   b. Where did you get this kind of information? Did you get information from clubs? What about from life skill training? From other ODA activities? From any other sources?
3. What do you think are the benefits of delaying marriage and first birth? (In terms of health, social, economic and education aspect). What do you think are the disadvantages of delaying marriage and first birth?
4. What do you think about girl's education? Do you think girls should go to school like boys do? What do you think will happen if you don't go to school any more now that you are married?
5. Up to what level should a girl continue her education before getting married? Why?

6. What is the highest level of education you would like to accomplish? And what kind of profession/job/ you would like to engage in? Up to what level should a girl continue her education before having her first baby?? Why?

7. I am informed that you are married. How old were you when you got married? Do you think this was a good marriage age? Why or why not?

8. Now that you are married, have you used any family planning method to delay your first birth? Do you have any children? How old is/are he/she/they? IF MORE THAN ONE CHILD, ASK: Did you use contraception to space your children?

9. Where did you receive information about contraception? IF USED CONTRACEPTION, ASK: Where did you get the method(s) you use/have used?

PART 2: CAUSES AND CONSEQUENCES OF EARLY MARRIAGE AND FIRST BIRTH

Next I would like to talk with you about the causes and consequences of early marriage and first birth.

1. Have you received any information about causes and consequence of early marriage and first birth?

- IF NO:
  - Why did you not receive this information?
  - Who do you think should provide this information? If you wanted this type of information, where would you get it?
- IF YES:
  - From whom did you receive the information? Did you get any information from ODA? Anyone else?
  - What type of information and services did you receive? From ODA? From others?
  - What information did you get about the cause and consequence of early marriage ? First birth? Delayed marriage

4. What did you think about the information you received? Were you satisfied or dissatisfied? Why?

5. What did other people think about you seeking information? Were they supportive? Was anyone against it? Who and why?
   - What does your family think about the information your got?? What does your husband’s family think? Community? Teachers? What do they think?
   - Did your husband receive any information about age of marriage, first birth and contraception? Whom did he get this information from? PROBE FOR INFO FROM THE RESPONDENT TO THE HUSBAND.

Now I would like to discuss using the following mapping exercise:

SOCIAL NETWORK (Mapping)

Note to interviewer: *You will use 2 pieces of paper with a "map" of concentric circles on each (see example below). The smallest, center circle will be marked as "I". Subsequent circles will be marked as family, peers, community members including health workers and people outside the community. Using the questions below you will ask the respondent to reflect upon the people who provide them with informational support (such as helping or advising) related to early marriage and first birth and individuals who pose as barriers. You or the respondent will*

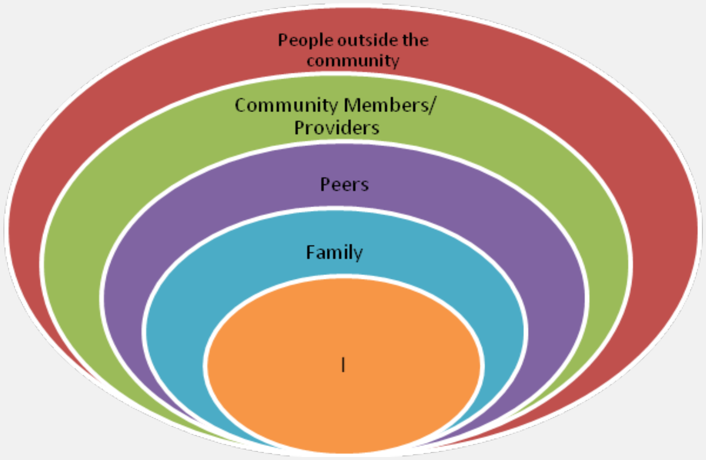

write each of these people into the appropriate circle.

I would now like to talk with you about whom you talk to and how you got information related to the decision for you to marry when you did. Let's start at the smallest circle. This circle is you. The other circles represent your family, peers, community members and health providers and people outside the community.

1. Now let's talk about people who support you by sharing information about early marriage and first birth, such as advising you on what to do?
   - Who in your family provides this support?
   - Peers?
   - Other people in the community? What about HEWs and health workers? Teachers?
   - People outside of the community?

4. Is there anywhere else you get information about delaying marriage and first birth or how to stay unmarried? For example: Radio or television?

   - Which of these people or other sources do you trust the most? Why?
   - Which of these people or other sources do you trust the least? Why?
   - If you wanted to delay marriage and first birth whose support would be most important to you? What are the reasons for this answer?

5. Now let's think about people who wanted you to get married when you did.
    - Who in your family wanted you to get married when you did? Your husband's family?
    - Peers?
    - Other people in the community? What about health workers? Health Extension workers?
    - People outside of the community?

PART 3: DECISION MAKING PROCESS

I would like to now talk about the decision for you to get married when you did.
How was the marriage proposal made, and by whom? And why was this individual proposed?

- Was the marriage proposal presented to you or did you hear about it through somebody else?
- If the proposal directly came to you, who told you about it first? Why?
  What was the age of the boy/man who proposed marriage?

1. Who in your family decided that you would get married when you did? What was your first reaction?
    - o How did you learn about their decision?
    - o Did you or your husband's families discuss with you the decision about getting married? Why/why not?
    - Who made the decision for you to get married?
    - How easy or difficult were these decisions? Why?

2. How did the decision maker(s) come to their decisions, and what role, if any, did the ODA project play in these decisions?

3. Were there other people who were influential in the decision? If so, who? How did they influence the decision?

4. Did you try to cancel or postpone your marriage? Who supported you? Who refuse your decision? Please briefly tell me about these events and what led to your family proceeding with the marriage?

5. Was there agreement within the family about the decision for you to marry when you did?
6. Who in your family had the final say about continuing with your marriage?
7. Were you happy with the decision? Why or why not? Who supported you during in the decision-making process?
8. What support did he/she/ they have given you after you got married?
9. Do you think the decision was right one? Why or why not?
10. How did the proposed groom's family react to the cancellation/postponement? Was there any pressure from the groom's family? What was your response and who supported you to stay unmarried?

PART 4: ENABLING FACTORS

First I would like to talk with you about enabling factors to continue with your marriage.

1. What were the factors that influenced (REFER TO THE DECISION MAKERS) to have you marry when you did/continue with the marriage?

2. Were there discussions with (DECISION MAKERS) about waiting for your marriage until you turned 18? If so, who had these talks with (DECISION MAKERS)? You? Teachers? ODA? Others?
3. How did (DECISION MAKERS) come to a decision to continue with the marriage?

4. What do you know about existing laws and regulations about early marriage? What do your parents know about these laws and regulations? What about your husband's parents?
   - How do laws and regulations affect someone like you?
   - How do you know about the existing laws and regulations on early marriage? Did ODA have something to do with it?

5. What was your experience with ODA interventions (sport for life, community awareness, and award provided to parents, saving for schooling, counseling, referral, girls club etc).
   - Do these activities help you to delay your marriage? Why not?

6. Then I would like to talk with the people who decided you should marry when you did. Can you tell me who decided that you should get married when you did? Why?

7. Of all the people who wanted you to marry when you did, who do you think are the most important ones? Can you tell me the one, two or three people who were the most important in deciding that you would marry when you did/your marriage would go forward? You don't have to name three people if only one or two made the decision.

8. I really want to speak to them, do you mind?

PART 5: PERCEPTION OF ODA ACTIVITIES (Free-listing)

In the last part of this interview, I would like to discuss your thoughts about ODA activities;

1. First, let's think about the ODA activities in your community regarding early marriage and first birth;
   - Can you describe what role ODA plays in the community in delaying marriage and first birth?
   - Can you tell me three words that would come to your mind if you were asked to describe ODA activities regarding girls' attitudes toward gender equality, early marriage and their perceived role in deciding when to marry?
   - Next, can you tell me three qualities that come to your mind about ODA's activities?

2. How girl’s participation in ODA activities did affected relationships with their parents? What did you benefit ODA activities help to improve your relationship with your parents?

3. Now, let’s think about ODA’s project to change the relationship between the girls and their parents.

   - Can you describe ODA’s role in changing the relationship between girls and parents in the community? If so, how? Were there challenges along the way?
   - Can you tell me three words that would come to your mind if you were asked to describe ODA’s activities regarding changing the relationship between girls and parents (Note: for example, is there any meaningful discussion between girls and parents).
   - Next, can you tell me three qualities that come to your mind about ODA’s activities in changing relationship between girls and parents?

PART 6: PERCEPTION OF SERVICE PROVIDERS (Free-listing)

In the last part of this interview, I would like to discuss your thoughts about local health workers and school teachers.

1. First, let’s think about your local health workers or health extension worker.

   - Can you describe what role these workers play in ODA intervention on early marriage and first birth?
   - Can you tell me three words that would come to your mind if you were asked to describe the service they have provided to you through their youth friendly health services (Note: if no care on youth friendly health service, ask about general care on SRH).
   - Next, can you tell me three qualities that come to your mind about this provider?

2. Now, let’s think about your local school teacher.

   - Can you describe what role your teachers and principal play in ODA interventions on early marriage and first birth?
   - Can you tell me three words that would come to your mind if you were asked to describe their regarding the SRH and youth friendly services they have provided; for example, separate toilets for boys and girls, the legal and healthy age for marriage and first birth? Other important information they gave you? Next, can you tell me three qualities that come to your mind when you think about your teachers and your principal?

CLOSING

Thank you very much for your time and for answering these questions. Before we end this interview, is there anything else you think we should discuss?

Thank you. I would like to assure you again that everything we have discussed today would be kept confidential.

In-Depth Interview

3. Parents, guardians or other individuals who made the decision to cancel or postpone the marriage

Socio-demographic

Step 1: Obtain consent from the respondent.
Step 2: Begin the interview.

Socio-demographic

Region______________Zone____________Woreda____________school/kebele___________
Age____ Educational level_________ Religion________________
HH owner___________ what is the relationship with her_________________. Family income source_____________________
Date of the interview____________ Start time __________end time _____________
Name of Interviewee____________________
Responsibility ________________________ Supervisor_________________

PART 1: KNOWLEDGE, ATTITUDE AND PERCEPTION ON EARLY MARRIAGE AND FIRST BIRTH

First I would like to talk with you about your opinions on the age at which girls should marry and have their first baby.

1. What does it mean to be a grown up as an adolescent in your locality? Can you tell me about (NAME'S) growing-up from childhood to date? When was the time she enjoyed the most? Was there a time when things with her got challenging? (probe until you get ample background information)

2. At what age do you think girls should get married? Do you think there are benefits and disadvantages to delaying marriage and first birth until at least age 18? Can you tell me any social, economic and health benefits of delaying marriage and first birth?

3. At what age you think girls should have their first baby? Can you tell me what you know about early marriage and first birth?
    - How are girls under age 18 affected by early marriage and first birth? You can talk about your own experiences if you'd like.
    - Where did you get your information about early marriage and childbirth? PROBE: PROBE: From (GIRL'S NAME)? From ODA? From health extension worker?

3. What do you think are the benefits of delaying marriage and first birth? (In terms of health, social, economic and education aspect). What do you think are the disadvantages of delaying marriage and first birth?

4. What do you think about girl's education? Do you think girls should go to school like boys do? What can happen if a girl doesn't go to school? Up to what level should a girl continue her education before getting married? Should a married girl stay in school?

5. How long do you think (GIRL'S NAME) should stay in school? Why? Did you go to school? If yes, until what age?

6. How and where do adolescents get information about the benefits of delaying marriage and first birth?

PART 2: CAUSES AND CONSEQUENCES OF EARLY MARRIAGE AND FIRST BIRTH

Next, I would like to talk with you about the causes and consequences of early marriage and first birth.

1. At what age most girls get married in your community? What are the reasons for girls to get married early before the age of 18?

2. What age is appropriate for girls to marry? What will happen if girls married before age of 18 describe the dangers of early marriage and childbirth?

3. How old were she when she proposed to got married?

4. How old were you when you had your first child? Was that a good age to become a mother? Did you have any health or economic problems from having a baby at that age?
5. Do you want (GIRL'S NAME) to have the same experience you had with getting married and having your children? Why or why not?

PART 3: DECISION MAKING PROCESS

Now I would like to talk about the decision to cancel/postpone the marriage.

1. First, how was the marriage proposal made, and by whom? And why was this individual proposed?
   - Was the marriage proposal presented to (GIRL'S NAME) or did she hear about it through somebody else?
   - If the proposal was directly to her, who did told her about it first? Why?
   - What was the age of the boy/man who proposed marriage to her?

2. Who first talked about the possibility of cancelling/postponing your marriage? Then what happened? Please briefly tell me the chain of events that eventually led to the marriage being cancelled/postponed.

3. Who made the final decision? If it was somebody else or more than one person, who was it? Describe their relationship to (GIRL'S NAME).
   - Do you usually discuss marriage and girl's education within the family? Did you discuss cancelation or postponement of the marriage? Was there agreement among all family members about the decision?
   - How easy or difficult were these decisions for you? And for her? For other family members? For the boy's/man's family? Why?

4. How did you/those of you who decided to cancel/postpone the marriage come to your/their decisions, and what role, if any, did the ODA project play in the decision?

5. Were there other people who were influential in the decision? If so, who? How did they influence the decision?

6. Why did she decide to cancel or postpone?

7. Who supported her in the decision process?

8. What support did you give to her?

9. How easy or difficult was the decision making?

10. Do you still think the decision was the right one?

11. How did the proposed groom's family react to the cancellation/postponement? Was pressure from the groom's family one of the reasons you continued with the early marriage?

12. Now that her marriage is canceled and/or postponed, is she still in touch with the person who had proposed to marry her? If yes, how do you describe their relationship?

PART 4: ENABLING FACTORS

Now I would like to talk with you about factors that helped you decide to cancel/postponed her marriage.

1. What enabled her to cancel and/or postpone the marriage?

2. What do you know about existing laws and regulations about early marriage?
   a. How do laws and regulations affect someone like her?
   b. Did ODA have anything to do with learning about the laws? With the marriage cancellation/postponement?

3. Can you tell me about any of the school factors (gender responsiveness), teachers that supported the decision to cancel/postpone her marriage?

4. Can you tell me about any community support that also supported the decision? For example, how supportive were other families, law enforcement bodies, and other in the decision to cancel/postpone her marriage?

5. Of all the people who supported her in the canceling/postponement of the marriage, who do you think were the most important ones?

PART 5: PERCEPTION OF ODA ACTIVITIES (Free-listing)

In the this part of this interview, I would like to discuss your thoughts about ODA activities;

1. First, let's think about ODA activities in your community regarding early marriage and first birth;
   a) Can you describe what role ODA plays in the community in delaying marriage and first birth?
   b) Can you tell me three words that would come to your mind if you were asked to describe ODA activities regarding girls' attitudes toward gender equality, early marriage and their perceived role in deciding when to marry?
   c) Next, can you tell me three qualities that come to your mind about ODA's activities?

2. Now, let's think about ODA's project to change the relationship between the girls and their parent's

   a) Can you describe ODA's role in changing the relationship between girls and parents in the community? If so, how? Were there challenges along the way?
   b) Can you tell me three words that would come to your mind if you were asked to describe ODA's activities regarding changing the relationship between girls and parents (Note: for example, is there any meaningful discussion between girls and parents).
   c) Next, can you tell me three qualities that come to your mind about ODA's activities in changing relationship between girls and parents?

PART 6: PERCEPTION OF SERVICE PROVIDERS (Free-listing)

In the last part of this interview, I would like to discuss your thoughts about local health workers and school teachers.

1. First, let's think about your local health workers or health extension worker.

   a) Can you describe what role these workers play in ODA interventions on early marriage and first birth?
   b) Can you tell me three words that would come to your mind if you were asked to describe this provider regarding the youth friendly services they have provided to adolescent girls (Note: if no care from youth friendly health services, ask about general care on SRH).
   c) Next, can you tell me three qualities that come to your mind about this provider?

3. Now, let’s think about her local school teacher and principal.

   a) Can you describe what role do these workers play in ODA intervention on early marriage and first birth?
   b) Can you tell me three words that would come to your mind if you were asked to describe teachers and the principal regarding the SRH and youth friendly service services they have provided (Note: for example, separate toilets for girls and boys, the appropriate age to get married).
   c) Next, can you tell me three qualities that come to your mind about school teachers and principals in your community?

PART 7 : FINDING SOLUTIONS

In the last few minutes, I would like to ask you what you think can be done to prevent early marriage and first birth in this community?

1) What can families/teachers/local leaders do? What would help them? What would make it difficult? What role does parents/teachers/local leaders/ODA have to play? What about other people in the family? What role do they play?

2) What about the role of the larger community? What can the community do to prevent early marriage and first birth among adolescent girls? What would help? What would make it difficult?

CLOSING

Thank you very much for your time and for answering these questions. Before we end this interview, is there anything else you think we should discuss?

Thank you. I would like to assure you again that everything we have discussed today would be kept confidential.

In-Depth Interview

4. Parents, guardians or other individuals who made the decision to proceed with marriage.

Step 1: Obtain consent from all respondents before beginning the interview.
Step 2: Begin interview.

Socio-demographic

Region______________Zone____________Woreda____________school/kebele___________
Age____ Educational level_________ Religion________________
HH owner_______ what is your relationship with Her _____________Family income source___________
Date of the interview____________ Start time __________end time _____________
Name of Interviewee____________________
Responsibility ________________________ Supervisor_________________

PART 1: KNOWLEDGE, ATTITUDE AND PERCEPTION ON EARLY MARRIAGE AND FIRST BIRTH

First I would like to talk with you about what your opinions about the age when girls should marry, and what you see as the benefits and disadvantages of early marriage..

1. What does it mean to be a grown up as an adolescent in your locality? Can you tell me about (GIRL'S NAME) growing-up from childhood to date? When was the time she enjoyed the most? When was it really got challenging? (probe until you get ample background information)

2. At what age you think girls should get married? Why? What is the benefit of delaying marriage and first birth until age 18? Can you tell me the social, economic, educational and health benefit of delaying marriage and first birth? What are the disadvantages of delaying marriage?

3. How and where did you get information about the benefits and disadvantages of delaying marriage and first birth until age 18?

4. At what age you think girls should have their first baby? Can you tell me what you know about early marriage and first birth?
   - How are adolescents affected by early marriage and first birth?
   - Where do adolescents get information about the benefits and disadvantages of marrying before age 18? Is it from clubs? Life skills training? Other activities?

7. What do you think about girl's education? Do you think girls should go to school like boys do? What happens if girls don't go to school?

8. Up to what level should a girl continue her education before getting married? Why?

PART 2: CAUSES AND CONSEQUENCES OF EARLY MARRIAGE AND FIRST BIRTH

Next, I would like to talk with you about the causes and consequences of early marriage and first birth.

1. At what age most girls get married in your community? What are the reasons for girls to get married early before the age of 18?

2. What age is appropriate for girls to marry? If you think girls should be married before age of 18 describe the importance or dangers of early childbirth. Yet you went ahead with an early marriage for the adolescent girl? Why?

3. How old were she when she got married? How old were you when you had your first baby? Do you think you were the right age when you married, were you too young, or too old? Why did you marry at that age?
4. How old were you when you had your first child? Was that a good age to become a mother? Did you have any health or economic problems from having a baby at that age?
5. Do you want (GIRL'S NAME) to have the same experience you had with getting married and having your children? Why or why not?

PART 3: DECISION MAKING PROCESS

I would like to now talk about the decision for (GIRL'S NAME) to get married.

1. How was the marriage proposal made, and by whom? And why was this individual proposed?
   - Was the marriage proposal presented to her or did she hear about it through somebody else?
   - If the proposal was directly to her, who did told her first? Why?

   What was the age of the boy/man who proposed marriage?

2. Who made the decision that she would get married when she did? What was her first reaction?
   - o How did she learn about the decision?
   - o Did you or other family members discuss with her the decision about getting married? Why/why not?
   - How easy or difficult were these decisions for her? Why?

3. How did you come to their decision? Was anyone from ODA involved as you made the decision?

4. Were there other people who were influential in the decision? If so, who? How did they influence the decision?

5. Was there agreement within the family about the decision for her to marry/to continue with her marriage? Was she happy about the decision?

6. Did she try to cancel or postpone the marriage? If yes, who supported her in trying to cancel or postpone the marriage? Did anyone from ODA talk with you or other family members about cancelling or postponing the marriage?

7. What support did he/she/they given her after she got married?

8. Do you still think the decision was right one? Why or why not?

PART 4: ENABLING FACTORS

Now I would like to talk with you about factors that influenced your decision for her to marry when she did.

1. What were the factors that made you think this was the right thing to do?

2. IF ATTEMPT MADE TO CANCEL OR POSTPONE MARRIAGE, ASK: What did you think about the reasons she and others gave for cancelling or postponing the marriage?

3. What do you know about existing laws and regulations about early marriage?
   - How do laws and regulations affect someone like her?
   - How do you know about the existing laws and regulations on early marriage? Did ODA have something to do with it?
   - What age do you think is the youngest that a girl should be legally allowed to marry?

4. Of all the people supported you in the decision to continuing with the marriage, who were the most important ones?

PART 5: PERCEPTION OF ODA ACTIVITIES (Free-listing)

In the last part of this interview, I would like to discuss your thoughts about ODA activities.

1. First, let’s think about the ODA activities in your community regarding early marriage and first birth;
   a) Can you describe what role ODA play’s in the community in delaying marriage and first birth until age 18?
   b) Can you tell me three words that would come to your mind if you were asked to describe ODA activities regarding girls’ attitudes toward gender equality, early marriage and their perceived role in deciding when to marry?
   c) Next, can you tell me three qualities that come to your mind about ODA’s activities?

d)

2. Now, let's think about ODA's project to change the relationship between the girls and their parents.

   a) Can you describe ODA's role in changing the relationship between girls and parents in the community? If so, how? Did (GIRL'S NAME) participation in ODA activities have any effect on your relationship with her in terms of discussing marriage, childbirth and contraception?
   b) Can you tell me three words that would come to your mind if you were asked to describe ODA's activities regarding changing the relationship between girls and parents (Note: for example, is there any meaningful discussion between girls and parents).
   c) Next, can you tell me three qualities that come to your mind about ODA's activities in changing relationship between girls and parents?

PART 6: PERCEPTION OF SERVICE PROVIDERS (Free-listing)

In the last part of this interview, I would like to discuss your thoughts about local health workers and school teachers and principals.

1. First, let's think about your local Health workers or health extension worker.

   a) Can you describe what role these workers play in ODA interventions on early marriage and first birth?
   b) Can you tell me three words that would come to your mind if you were asked to describe this provider regarding the youth friendly services they have provided to adolescents in your community (Note: if no care from youth friendly health services, ask about general care on SRH).
   c) Next, can you tell me three qualities that come to your mind about this provider?

2. Now, let's think about local school teachers and principals in your community.

   a) Can you describe what role teachers and principals play in ODA interventions on early marriage and first birth?
   b) Can you tell me three words that would come to your mind if you were asked to describe teachers in your community regarding the SRH and youth friendly services they have provided (Note: for example, separate toilets for girls and boys, the appropriate age at which to get married).
   c) Next, can you tell me three qualities that come to your mind about this provider?

PART 7 : FINDING SOLUTIONS

In the last few minutes, I would like to ask you what you think can be done to prevent early marriage and first birth in this community?

3) What can families/teachers/local leaders do? What would help them? What would make it difficult? What role does parents/teachers/local leaders/ODA have to play? What about other people in the family? What role do they play?

4) What about the role of the larger community? What can the community do to prevent early marriage and first birth among adolescent girls? What would help? What would make it difficult?

CLOSING

Thank you very much for your time and for answering these questions. Before we end this interview, is there anything else you think we should discuss?

Thank you. I would like to assure you again that everything we have discussed today would be kept confidential.

Interview Guides: India

In-Depth Interview

1. Women who participated in RISHTA activities and whose marriages before age 18 were cancelled or postponed.

Step 1: Obtain consent from all respondents before beginning the interview.
Step 3: Begin the interview

Socio-demographic

Village____

Age____ Highest Education level_________ Religion________________

HH owner____________ What is your birth order?_____ child in the family.

Main source of family income ____

Date of the interview____________ Start time __________end time _____________

Name of respondent _____________________________________ Respondent #___________

Name of interviewer______________________________________ Interviewer #___________

PART 1: KNOWLEDGE, ATTITUDE AND PERCEPTION ON EARLY MARRIAGE AND FIRST BIRTH

First I would like to talk with you about your opinions of the age when girls should marry, and what you see as the benefits and disadvantages of marrying before age 18.

1. What do you think it means in your community/village to be a grownup while you are still an adolescent? Can you tell me about your childhood from when you were very young to now? What was the period you enjoyed the most? Have you had times that were challenging? (PROBE FOR AMPLE BACKGROUND INFORMATION.)

2. At what age do you think girls should get married? What are the benefits and disadvantages of delaying marriage until at least age 18? Can you tell me the social, economic, health and educational benefits or disadvantages of delaying marriage? Others?

3. At what age you think girls should have their first baby?
   a. How do you think it would affect someone like you to have a baby before age 18?
   b. Where did you get this information? (PROBE)

4. What do you think about girls' education? Do you think girls should go to school like boys do? Do you think the future is different for girls who go to school compared with those who don't? If so, in what ways?

5. Up to what level should a girl continue her education before getting married? Why?

6. IF EDUCATION NOT COMPLETE, ASK: What is the highest level of education that you would like to complete? And what kind of profession or job would you like to have?
7. Up to what level should a girl continue her education before having her first baby? Why?

PART 2: CAUSES AND CONSEQUENCES OF EARLY MARRIAGE AND FIRST BIRTH

Next I would like to talk with you about the causes and consequences of early marriage
.

1. At what age do most girls get married in your village? What are the reasons that girls get married before the age of 18?

2. What did you think about the information you have received from Project RISHTA on the benefits of waiting until 18 years to marry? Were you satisfied or dissatisfied with the information you received? Why?

3. What did other people think about your participation in Project RISHTA? Were they supportive? Was anyone against it? Why?

4. What about your immediate family members? What did they think about RISHTA? What did your brothers and sisters think? What about your friends?

Now I would like to discuss using the following mapping exercise:

SOCIAL NETWORK (Mapping)
Note to interviewer: *You will use 2 pieces of paper with a "map" of concentric circles on each (see example below). The smallest, center circle will be marked as "I". Subsequent circles will be marked as family, peers, community members (including RISHTA people), and people*

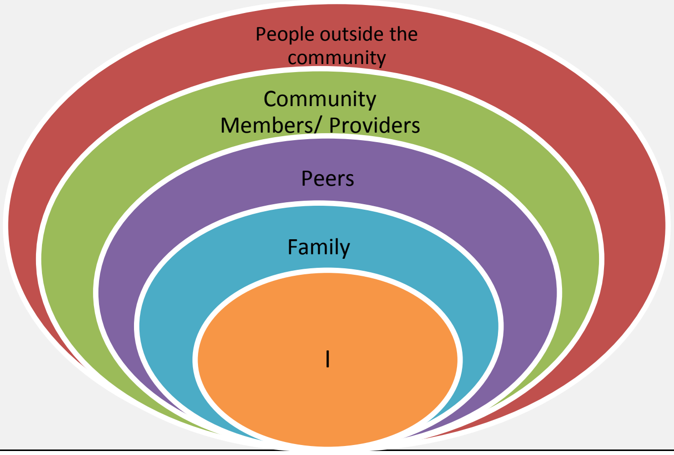

outside the community. She can name the outsiders. Using the questions below you will ask the respondent to reflect upon the people who supported them in learning about early marriage, and those who opposed it. You or the respondent will write each of these people into the appropriate circle.

Now I would like to talk with you about how the information you received influenced the cancellation/postponement of your marriage. Let's start at the smallest circle. This circle is you. The other circles represent your family, peers, Project RISHTA people and other community members, and people outside the community.

1. Let's talk about people who supported you in obtaining information about early marriage and first birth?
   a. Who in your family provided this support?
   b. Peers?
   c. Other people in the community? YRCs? Other RISHTA people?
   d. People outside of the community? (PROBE FOR WHO THEY ARE AND THEIR RELATIVE IMPORTANCE IN OBTAINING INFORMATION.)

2. Is there anywhere else you got information about delaying marriage and or how to stay unmarried until you are at least 18 years of age? For example: Radio or television?

3. Now let's think about people who tried to make the marriage happen before age 18.
   a. Were there family members who pushed for the marriage? Who?
   b. Peers?
   c. Other people in the community?
   d. People outside of the community? (PROBE FOR WHO AND RELATIVE IMPORTANCE.)

4. Thinking about all of these people that we just talked about,
   a. Which of these people do you trust the most? Why?
   b. Which of these people do you trust the least? Why?

PART 3: DECISION MAKING PROCESS

Now let's talk about the decision to cancel/postpone your marriage.

1. First, how was the marriage proposal made, and by whom? Why was this groom chosen?
   a. Was the marriage proposal presented to you or did you hear about it through somebody else?
   b. If the proposal directly came to you, who told you about it first?
   c. What was the age of the boy/man who proposed marriage to you?

2. Who first talked about the possibility of cancelling/postponing your marriage—you or someone else? Then what happened? Please briefly tell me the chain of events that eventually led to the marriage being cancelled/postponed.

3. Who made the final decision? IF IT WAS NOT THE GIRL'S, ASK:

    a. How did you learn of their decision?
    b. Do/Did you usually discuss marriage and girls' education within your family? Did you discuss cancelation and postponement of the marriage? Was there agreement among all family members about the decision?
    c. If postponed, do you still have a relationship with this man/Is this the man you ultimately married? If yes, how would you describe your relationship now? Do have a sexual relationship? Do you use any protection or family planning method? Where did you get the family planning method?
    d. How easy or difficult were all of these decisions for you and your family? Why?

4. How did the decision maker(s) come to their decisions, and what role, if any, did the YRCs/Project RISHTA play in the decision?

5. Were there other people who were influential in the decision? If so, who? How did they influence the decision?

6. IF THE WOMAN DECIDED, ASK: Who supported you in your decision process, and in what ways?

7. What support did he/she/they give you?

8. How easy or difficult was the decision for you?

9. Who in your family usually has the final say on marriage decisions?

10. Do you still think the decision was the right one?

PART 4: ENABLING FACTORS

1. What were the factors that contributed to the decision to cancel/postpone the marriage?

2. What do you know about existing laws regarding early marriage?
    a. How do these laws affect someone like you?
    b. How did you learn about these existing laws?
    c. Did Project RISHTA have something to do with it?

3. Can you tell me about community support you received for the decision to cancel/postpone your marriage? For example, how supportive were other families, law enforcement bodies, women's self-help groups, panchayat, teachers, others?

4. How did your proposed groom react to the cancellation/postponement? How did his family react?

5. What was your experience with Project RISHTA? Did RISHTA activities help you to cancel/postpone your marriage? How?

6. IF NOT IN A RELATIONSHIP WITH THE PROPOSED GROOM, ASK: Are you now married/engaged to someone else? In a romantic relationship with someone else? IF NOT MARRIED, Are you involved in a sexual relationship with this person? IF MARRIED OR IN SEXUAL RELATIONSHIP BUT NOT MARRIED, ASK: Do you use any contraceptive? Where do you get the contraceptives?

Next, I would like to talk with the people who made the decision to cancel/postpone your marriage. I know this was a big decision for your family, and we are interested in hearing from these people regarding how they came to make this decision.

7. Of all the people who supported you in canceling/postponing the marriage, who do you think were the most important ones? Can you tell me the one, two or three people you think that, without their support, things would have ended differently? You don't have to name three people if only one person or two made the decision.

PART 5: PERCEPTION OF PROJECT RISHTA ACTIVITIES (Free-listing)

In the last part of this interview, I would like to discuss your thoughts about Project RISHTA activities;

1. First, let's think about the Project RISHTA activities in your community regarding early marriage.
   a. Can you describe the Project RISHTA activities that influenced the community about delaying marriage until at least age 18?
   b. Can you tell me three words that come to mind to describe RISHTA activities related to girls' attitudes toward gender equality, early marriage and their perceived role in deciding when to marry? (PROBE IF NEEDED)
   c. Next, can you tell me three qualities that you would use to describe RISHTA activities in general?

2. How do you think your participation in RISHTA affected your relationship with your parents?

3. Can you describe the role that the YRCs played in cancelling or delaying your marriage?

4. Can you tell me three words that come to mind to describe the services that the YRCs have provided to you?

PART 6 : FINDING SOLUTIONS

In the last few minutes, I would like to ask you what you think can be done to prevent early marriage and first birth in this community?

1. What can families, local leaders and you do? What would help them? What would make it difficult? What role does Project RISHTA have to play?

2. What about the role of the larger community? What can the community do to prevent early marriage and first birth among adolescent girls? What would help? What would make it difficult?

CLOSING

Thank you very much for your time and for answering these questions. Before we end this interview, is there anything else you think we should discuss?

Thank you. I would like to assure you again that everything we have discussed today will be kept confidential.

In-Depth Interview
2. Participating young women who were married before age 18

Step 1: Obtain consent from all respondents before beginning the interview.
Step 2: Begin the interview.

Socio-demographic

Village____

Age____ Highest Education level_________ Religion________________

HH owner____________ What is your birth order?_____ child in the family.

Main source of family income ____

Date of the interview____________ Start time __________end time _____________

Name of respondent ____________________________________ Respondent #___________

Name of interviewer______________________________________ Interviewer #___________

PART 1: KNOWLEDGE, ATTITUDE AND PERCEPTION ON EARLY MARRIAGE AND FIRST BIRTH

First I would like to talk with you about your opinions on the benefits and disadvantages of marrying before age 18.

1. What do you think it means in your community/village to be a grownup while you are still an adolescent? Can you tell me about your childhood from when you were very young to now? What was the period you enjoyed the most? Have you had times that were challenging? (PROBE FOR AMPLE BACKGROUND INFORMATION.)

2. At what age do you think girls should get married? What are the benefits and disadvantages of delaying marriage until at least age 18? Can you tell me the social, economic, health and educational benefits or disadvantages of delaying marriage? Others?

3. At what age you think girls should have their first baby?
    a. How do you think it would affect someone like you to have a baby before age 18?
    b. Where did you get this information? (PROBE)

4. What do you think about girls' education? Do you think girls should go to school like boys do? Do you think the future is different for girls who go to school compared with those who don't? If so, in what ways?

5. Up to what level should a girl continue her education before getting married? Why?

6. Up to what level should a girl continue her education before having her first baby? Why?

7. IF EDUCATION NOT COMPLETE, ASK: What is the highest level of education that you would like to complete? And what kind of profession or job would you like to have?

8. I am informed that you are married. How old were you when you got married? Do you think this was a good marriage age? Why or why not?

9. Now that you are married, have you used any family planning method to delay your first birth? Do you have any children? How old is/are he/she/they? IF MORE THAN ONE CHILD, ASK: Did you use contraception to space your children?

10. Where did you receive information about contraception? IF USED CONTRACEPTION, ASK: Where did you get the method(s) you use/have used?

PART 2: CAUSES AND CONSEQUENCES OF EARLY MARRIAGE AND FIRST BIRTH

1. Before you were married, did you receive any information about the disadvantages and benefits of marrying before age 18?
   IF NO:
   a. Who do you think should provide this information?
   b. If you wanted this type of information, where would you get it?

   IF YES:
   a. From whom did you receive the information? Did you get any information from Project RISHTA? Anyone else?
   b. What did you learn? From RISHTA? From others?
   ~~c.~~ Did you learn about the appropriate age to have your first baby?

2. What did you think about the information you received? Were you satisfied or dissatisfied? Why?

3. What did other people think about you seeking information? Were they supportive? Was anyone against it? Who and why?
   a. What does your family think about the information you got? What does your husband's family think? Community members?

b. Did your husband receive any information about age of marriage, first birth and contraception before your marriage? Whom did he get this information from? PROBE FOR INFO FROM THE RESPONDENT TO THE HUSBAND.

Now I'd like to do a mapping exercise with you:

SOCIAL NETWORK (Mapping)

Note to interviewer: *You will use 2 pieces of paper with a "map" of concentric circles on each (see example below). The smallest, center circle will be marked as "I". Subsequent circles will be marked as family, peers, community members (including RISHTA people), and people outside the community. Using the questions below you will ask the respondent to reflect upon the people who supported them in learning about early marriage, and those who opposed it.*

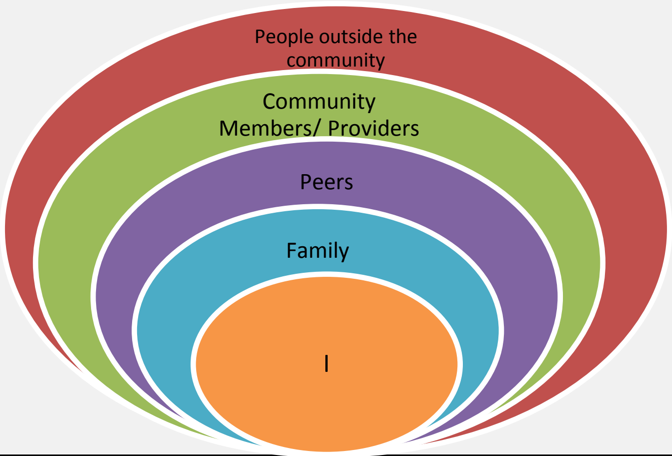

Now I would like to talk with you about how the information you received influenced the decision for you to marry when you did. Let's start at the smallest circle. This circle is you. The other circles represent your family, peers, Project RISHTA people and other community members, and people outside the community.

1. Let's talk about people who supported you in obtaining information about early marriage and first birth?
   a. Who in your family provided this support?
   b. Peers?
   c. Other people in the community? YRCs? Other RISHTA people?
   d. People outside of the community?

2. Is there anywhere else you got information about delaying marriage and or how to stay unmarried until you are at least 18 years of age? For example: Radio or television?

3. Now let's think about people who wanted you to get married when you did.
   a. Who in your family wanted you to get married when you did? What about your husband's family?

b. Peers?
c. Other people in the community?
d. People outside of the community?

4. Thinking about all of these people that we just talked about,
   a. Which of these people do you trust the most? Why?
   b. Which of these people do you trust the least? Why?
   c. Whose support was most important to you? Why?

PART 3: DECISION MAKING PROCESS

1. How was the marriage proposal made, and by whom? Why was this man chosen? IF LOVE MARRIAGE BEFORE AGE 18, SKIP TO Q3.

2. Was the marriage proposal presented to you or did you hear about it through somebody else? If the proposal came directly to you, who told you about it first?

3. What was the age of the groom? IF LOVE MARRIAGE, SKIP TO Q4.

   a. Who in your family decided that you would get married when you did? What was your first reaction?
   b. How did you learn about their decision?
   c. Did you or your husband's family discuss the decision with you? Why/why not?

4. How easy or difficult was the decision for you? Why? IF LOVE MARRIAGE BEOFRE AGE 18, ASK: What did your family think about the marriage?

5. How did the decision maker(s) come to their decisions, and what role, if any, did Project RISHTA play in these decisions? IF LOVE MARRIAGE, ASK: Did anyone from Project RISHTA encourage you to wait until age 18 to marry?

6. Were there other people who were influential in the decision? If so, who? How did they influence the decision?

7. Did you try to cancel or postpone your marriage? IF YES, Who supported you? Who refused your wishes? Please briefly tell me about these events and what led to your family proceeding with the marriage? IF LOVE MARRIAGE, ASK: Tell me about the events that led to your marriage. Was there another groom that your family wanted you to marry?

8. Was there agreement within the family about the decision for you to marry when you did? IF LOVE MARRIAGE, SKIP TO Q10.

9. Who in your family had the final say about continuing with your marriage?

10. Were you happy with the/your decision? Why or why not? Who supported you during in the decision-making process? IF LOVE MARRIAGE, ASK: Did anyone support you in this marriage? Who and how?

11. What support has/have he/she/they given you since you got married?

12. Do you think the decision was the right one? Why or why not? IF LOVE MARRIAGE, ASK: Has your family accepted this marriage?

PART 4: ENABLING FACTORS

1. We talked about some of the reasons that your family/you decided to marry when you did. Are there any other factors that influenced (REFER TO THE DECISION MAKERS/YOU) in the decision to marry when you did?

2. Were there discussions with (DECISION MAKERS) about waiting for your marriage until you turned 18? If so, who had these talks with (DECISION MAKERS)? You? YRC members? Others?

3. How did (DECISION MAKERS/YOU) come to a decision to continue with the marriage? Why was it important for them/him/her/you that you marry when you did?

4. What do you know about existing laws regarding the age of marriage in India? What do your parents know about these laws? What about your husband's parents?
    a. How do the laws affect someone like you?
    b. How did you learn about the laws? (PROBE FOR RISHTA's PART.)

5. Of all the people who wanted you to marry when you did, who do you think were the most important ones? Can you tell me the one, two or three people who were the most important in deciding that you would marry when you did/your marriage would go forward? You don't have to name three people if only one or two made the decision. FOR LOVE MARRIAGES, ASK: Who were the people who most supported this marriage, besides you and your husband? Anyone? Who?

PART 5: PERCEPTION OF PROJECT RISHTA ACTIVITIES (Free-listing)

In the last part of this interview, I would like to discuss your thoughts about Project RISHTA activities.

1. First, let's think about the Project RISHTA activities in your community regarding early marriage.
    a. Can you describe the Project RISHTA activities that influenced the community about delaying marriage until at least age 18?
    b. Can you tell me three words that come to mind to describe RISHTA activities related to girls' attitudes toward gender equality, early marriage and their perceived role in deciding when to marry? (PROBE IF NEEDED)
    c. Next, can you tell me three qualities that you would use to describe RISHTA activities in general?

2. How do you think your participation in RISHTA affected your relationship with your parents?

3. Can you describe the role that the YRCs played in cancelling or delaying your marriage?

4. Can you tell me three words that come to mind to describe the services that the YRCs have provided to you?

PART 6 : FINDING SOLUTIONS

In the last few minutes, I would like to ask you what you think can be done to prevent early marriage and first birth in this community?

1. What can families, local leaders and you do? What would help them? What would make it difficult? What role does Project RISHTA have to play?

2. What about the role of the larger community? What can the community do to prevent early marriage and first birth among adolescent girls? What would help? What would make it difficult?

CLOSING

Thank you very much for your time and for answering these questions. Before we end this interview, is there anything else you think we should discuss?

Thank you. I would like to assure you again that everything we have discussed today will be kept confidential.

In-Depth Interview

3. Parents, guardians or other individuals who made the decision to cancel or postpone the marriage

Socio-demographic

Step 1: Obtain consent from the respondent.
Step 2: Begin the interview.

Socio-demographic

Village____

Age____ Highest Education level_________ Religion________________

HH owner____________ What is your birth order?_____ child in the family.

Main source of family income ____

Date of the interview____________ Start time __________end time _____________

Name of respondent _____________________________________ Respondent #___________

Name of interviewer______________________________________ Interviewer #___________

PART 1: KNOWLEDGE, ATTITUDE AND PERCEPTION ON EARLY MARRIAGE AND FIRST BIRTH

First I would like to talk with you about your opinions on the age at which girls should marry and have their first baby.

1. What does it mean to be considered a grown up in your community/village when still an adolescent? Can you tell me about (GIRL' S NAME) childhood from when she was very young until now? What was the period you think she enjoyed the most? Was there a time when things were challenging for her or when she was challenging? (probe until you get ample background information)

2. At what age do you think girls should get married?

3. At what age do you think women should have their first baby?
   a. How are girls under age 18 affected by early marriage and first birth? You can talk about your own experiences if you'd like.

b. Where did you get your information about early marriage and childbirth? PROBE: PROBE: From (GIRL'S NAME)? From Project RISHTA? Other sources?

4. What do you think are the benefits of delaying marriage until age 18? (In terms of health, social, economic and education aspect). What do you think are the disadvantages of delaying marriage and first birth?

5. What do you think about girls' education? Do you think girls should go to school like boys do? Do you think the future is different for girls who go to school compared with those who don't? If so, in what ways?

6. Up to what level should a girl continue her education before getting married? Should a married girl stay in school?

7. Did you go to school? If yes, until what age? IF PRIMARY RESPONDENT STILL IN SCHOOL, ASK: How long do you think (GIRL'S NAME) should stay in school? Why?

8. How and where do adolescents get information about the benefits of delaying marriage until at least age 18?

PART 2: CAUSES AND CONSEQUENCES OF EARLY MARRIAGE AND FIRST BIRTH

Next, I would like to talk with you about the causes and consequences of marrying before age 18.

1. At what age most girls get married in your village? What are the reasons that girls get married before the age of 18?

2. How old was (GIRL'S NAME) when marriage was proposed?

3. FOR FEMALE RESPONDENTS: How old were you when you got married? Had your first child? Was that a good age to become a mother? Did you have any health or economic problems from having a baby at that age?

4. Do you want (GIRL'S NAME) to have the same experience you had with getting married and having your children? Why or why not?

PART 3: DECISION MAKING PROCESS

Now I would like to talk about the decision to cancel/postpone (GIRL'S NAME) marriage.

1. First, how was the marriage proposal made, and by whom? And why was this groom chosen?

    a. Was the marriage proposal presented to (GIRL'S NAME) or did she hear about it through somebody else?
    b. If the proposal was not made directly to her, who told her about it first? Why?
    c. What was the age of the groom?

2. Who first talked about the possibility of cancelling/postponing the marriage? Then what happened? Please briefly tell me the chain of events that eventually led to the marriage being cancelled/postponed.

3. Who made the final decision? If it was somebody other than you, or if more than one person made the decision, who was it? Describe their relationship to (GIRL'S NAME).
    a. Do you usually discuss marriage and girls' education within your family? Did you discuss cancelation or postponement of the marriage? Was there agreement among family members about the decision?
    b. How easy or difficult was the decision for you? And for her? For other family members? For the groom's family? Why?

4. How did you/those of you who decided to cancel/postpone the marriage come to the decision, and what role, if any, did Project RISHTA play in the decision?

5. Were there other people who were influential in the decision? If so, who? How did they influence the decision?

6. Did (GIRL'S NAME) want to cancel or postpone the marriage? IF NO, PROBE WHY IT WAS CANCELLED.

7. If yes, who supported her in the decision making process?

8. What support did you give to her?

9. How easy or difficult was the decision for your family?

10. Do you still think the decision was the right one?

11. How did the proposed groom's family react to the cancellation/postponement?

PART 4: ENABLING FACTORS

Now I would like to talk with you about factors that helped you decide to cancel/postponed her marriage.

1. What were the main factors that led you to cancel/postpone the marriage? Did YRC or other RISHTA members play a role in cancelling or postponing the marriage?

2. What do you know about existing laws concerning the age of marriage in India?
   a. How do laws affect someone like (GIRL'S NAME)?
   b. Did Project RISHTA give you information about the legal age of marriage in India?

3. Can you tell me about any other community members who also supported the decision? For example, how supportive were other families, law enforcement bodies, and others in the decision to cancel/postpone her marriage?

4. Of all the people who supported the cancellation/postponement of the marriage, who do you think were the most important ones?

PART 5: PERCEPTION OF PROJECT RISHTA ACTIVITIES (Free-listing)

Next, I would like to discuss your thoughts about Project RISHTA activities.

1. First, let's think about the Project RISHTA activities in your village regarding early marriage.
   a. Can you describe what role Project RISHTA played in how people in your village think about early marriage?
   b. Can you tell me three words that come to mind in describing RISHTA activities regarding girls' attitudes toward gender equality, early marriage and their perceived role in deciding when to marry?
   c. Next, can you tell me three qualities that you would use to describe RISHTA activities in general?

2. How do you think your (GIRL'S NAME) participation in RISHTA affected her relationships with you/her parents?

3. Can you describe what role the YRCs played in delaying marriage until age 18?

4. Can you tell me three words that come to your mind in describing the services that the YRCs provide?

PART 6 : FINDING SOLUTIONS

In the last few minutes, I would like to ask you what you think can be done to prevent early marriage and first birth in this community?

1. What can families, local leaders and you do? What would help them? What would make it difficult? What role does Project RISHTA have to play?

2. What about the role of the larger community? What can the community do to prevent early marriage and first birth among adolescent girls? What would help? What would make it difficult?

CLOSING

Thank you very much for your time and for answering these questions. Before we end this interview, is there anything else you think we should discuss?

Thank you. I would like to assure you again that everything we have discussed today will be kept confidential.

In-Depth Interview

4. Parents, guardians or other individuals who made the decision to proceed with marriage.

Step 1: Obtain consent from all respondents before beginning the interview.
Step 2: Begin interview.

Socio-demographic

Village____

Age____ Highest Education level_________ Religion________________

HH owner____________ What is your birth order?_____ child in the family.

Main source of family income ____

Date of the interview____________ Start time __________end time _____________

Name of respondent _____________________________________ Respondent #___________

Name of interviewer______________________________________ Interviewer #___________

PART 1: KNOWLEDGE, ATTITUDE AND PERCEPTION ON EARLY MARRIAGE AND FIRST BIRTH

First I would like to talk with you about your opinions on the age at which girls should marry and have their first baby.

1. What does it mean to be considered a grown up in your community/village when still an adolescent? Can you tell me about (GIRL' S NAME) childhood from when she was very young until now? What was the period you think she enjoyed the most? Was there a time when things were challenging for her or when she was challenging? (probe until you get ample background information)

2. At what age do you think girls should get married?

3. At what age do you think women should have their first baby?
    a. How are girls under age 18 affected by early marriage and first birth? You can talk about your own experiences if you'd like.
    b. Where did you get your information about early marriage and childbirth? PROBE: PROBE: From (GIRL'S NAME)? From Project RISHTA? Other sources?

4. What do you think are the benefits of delaying marriage until age 18? (In terms of health, social, economic and education aspect). What do you think are the disadvantages of delaying marriage and first birth?

5. What do you think about girls' education? Do you think girls should go to school like boys do? Do you think the future is different for girls who go to school compared with those who don't? If so, in what ways?

6. Up to what level should a girl continue her education before getting married? Should a married girl stay in school?

7. Did you go to school? If yes, until what age? IF (PRIMARY RESPONDENT STILL IN SCHOOL, ASK: How long do you think (GIRL'S NAME) should stay in school? Why?

8. How and where do adolescents get information about the benefits of delaying marriage until at least age 18?

PART 2: CAUSES AND CONSEQUENCES OF EARLY MARRIAGE AND FIRST BIRTH

Next, I would like to talk with you about the causes and consequences of marrying before age 18.

1. At what age do most girls get married in your community? What are the reasons that girls marry before the age of 18?

2. What age do you think is appropriate for girls to marry? IF RESPONDENT SAYS AN AGE YOUNGER THAN 18, ASK: What are the advantages of marrying before age 18? What are the disadvantages? IF MENTIONS DISADVANTAGES, ASK What made you decide to proceed with an early marriage for (GIRL'S NAME)?

3. IF FEMALE, ASK: How old were you when you got married? Do you think you were the right age when you were married, were you too young, or too old? Why did you marry at that age?

4. How old were you when you had your first child? Was that a good age to become a mother? Did you have any health or economic problems from having a baby at that age?

5. Do you want (GIRL'S NAME) to have the same experience you had with getting married and having children? Why or why not?

PART 3: DECISION MAKING PROCESS

Now I would like to talk about the decision for (GIRL'S NAME) to marry when she did.

1. First, how was the marriage proposal made, and by whom? Why was this groom chosen? RESPONDENT SAYS THAT IT WAS A LOVE MARRIAGE, SKIP TO c.
    a. Was the marriage proposal presented to (GIRL'S NAME) or did she hear about it through somebody else?
    b. If the proposal was not made directly to her, who did told her about it first? Why?
    c. What was the age of the groom?
    d. Do you usually discuss marriage and girls' education within the family? Did you discuss (GIRL'S NAME) marriage? Was there agreement among all family members about the decision for her to marry when she did?
    e. How easy or difficult was the decision for you? And for her? For other family members? For the groom's family? Why?

2. Did anyone, including (GIRL'S NAME), talk about the possibility of cancelling or postponing the marriage until she turned 18? IF NO, SKIP TO 3. IF YES, ASK: Who talked about the possibility of cancelling/postponing the marriage? Then what happened? Please briefly tell me the chain of events that eventually led to the marriage.

3. Did (GIRL'S NAME) want to cancel or postpone the marriage? IF YES, Who supported her in wanting to cancel or postpone? Did you? IF SO, What support did you give to her?

4. Did anyone from the YRC or Project RISHTA try to have the marriage cancelled or postponed? IF YES, What did they do? What did you think about that?

5. Were there other people who tried to influence the decision one way or the other? If so, who? How did they influence the decision?

6. Who made the final decision for the marriage to occur when it did? If it was somebody other than you, or if more than one person made the decision, who was it—for example, the groom's family? Describe their relationship to (GIRL'S NAME).

7. Do you think the decision was the right one?

PART 4: ENABLING FACTORS

Now I would like to talk with you about factors that influenced your decision for her to marry when she did.   IF LOVE MARRIAGE, SKIP TO 3.

1. What were the factors that made you think this was the right thing to do?

2. IF ATTEMPT MADE TO CANCEL OR POSTPONE MARRIAGE, ASK: What did you think about the reasons she and others gave for cancelling or postponing the marriage?

3. What do you know about existing laws regarding the legal age of marriage in India?
    a. How do laws affect someone like her?
    b. How do you know about the laws? Did Project RISHTA have anything to do with it?
    c. What age do you think is the youngest that a girl should be legally allowed to marry?

4. Of all the people who wanted the marriage to take place when it did, who were the most important ones?

PART 5: PERCEPTION OF PROJECT RISHTA ACTIVITIES (Free-listing)

Next, I would like to discuss your thoughts about Project RISHTA activities.

5. First, let's think about the Project RISHTA activities in your village regarding early marriage.
    d. Can you describe what role Project RISHTA played in how people in your village think about early marriage?
    e. Can you tell me three words that come to mind in describing RISHTA activities regarding girls' attitudes toward gender equality, early marriage and their perceived role in deciding when to marry?
    f. Next, can you tell me three qualities that you would use to describe RISHTA activities in general?

6. How do you think your (GIRL'S NAME) participation in RISHTA affected her relationships with you/her parents?

7. Can you describe what role the YRCs played in delaying marriage until age 18?

8. Can you tell me three words that come to your mind in describing the services that the YRCs provide?

PART 6 : FINDING SOLUTIONS

In the last few minutes, I would like to ask you what you think can be done to prevent early marriage and first birth in this community?

1. What can families, local leaders and you do? What would help them? What would make it difficult? What role does Project RISHTA have to play?

2. What about the role of the larger community? What can the community do to prevent early marriage and first birth among adolescent girls? What would help? What would make it difficult?

CLOSING

Thank you very much for your time and for answering these questions. Before we end this interview, is there anything else you think we should discuss?

Thank you. I would like to assure you again that everything we have discussed today will be kept confidential.
